# Supplementary material for: Heterologous Expression of Secondary Metabolite Genes in Trichoderma reesei for Waste Valorization
Source: J Fungi (Basel). 2022 Mar 30;8(4):355. doi: 10.3390/jof8040355 (PMC9032437; doi:10.3390/jof8040355)
Supplement: Supplementary file 1 [file jof-08-00355-s001.zip › jof-1651595-supplementary.pdf]

Journal of *Fungi*

Article

Heterologous expression of secondary metabolite genes in *Trichoderma reesei* for waste valorization

Mary L. Shenouda [1,2], Maria Ambilika [1] Elizabeth Skellam [1,3] and Russell J. Cox [1,*]

1 Institute for Organic Chemistry and BMWZ, Schneiderberg 38, 30167, Hannover, Germany.
2 Pharmacognosy Department, Faculty of Pharmacy, Alexandria University, Alexandria, 21521, Egypt
3 Department of Chemistry and BioDiscovery Institute, University of North Texas, 1155 Union Circle, Denton, TX 76201, USA
* Correspondence: russell.cox@oci.uni-hannover.de

Electronic Supplementary Information

Contents

1. **Growth Media, Buffers, Solutions, Enzymes and Antibiotics** 2
2. **Bacterial and Fungal Strains** 5
3. **Vectors and Oligonucleotides** 6
4. **Microbiological Methods.** 8
5. **Molecular Biology Methods** 10
6. **Chemical Analysis** 12
7. **Vector Construction** 14
8. **Construction of a *T. reesei* QM6A *Δtmus53 Δpyr4* Sorbicillin Deletion Strain** 18
9. **Expressing the PKS *aspks1* in *T. reesei*.** 24
10. **Expressing the PKS-NRPS *tenS* and the ER *tenC* in *T. reesei*.** 33
11. **Selected NMR Spectra** 39
12. **References** 45

1. Growth media, buffers and solutions, enzymes and antibiotics

Table S1.1: Growth media:

Media	Composition [% (*w/v*)]	Ingredients
**GNB**	2.00	D(+)-Glucose monohydrate
	3.00	Nutrient broth Nr. 2 from Oxoid
**LB**	0.50	Yeast extract
	1.00	Tryptone
	0.50	NaCl
**SOB**	2.00	Tryptone
	0.50	Yeast Extract
	8.56 mM	NaCl
	2.50 mM	KCl
	10.0 mM	$MgCl_2$
**SOC**	93.75 (*v/v*)	**SOB**
	1.25	$MgCl_2 \cdot H_2O$
	5.00 (*v/v*)	D(+)-glucose 20 %
**YPAD**	1	Yeast extract
	2	Tryptone
	2	D(+)-Glucose Monohydrate
	0.03	Adenine
**ME(BF)**	2.00	Malt extract
	0.10	Peptone from Soya
	2.00	D(+)-Glucose monohydrate
**Mandels Andreotti (MA) + 1 % glucose**	0.14	$(NH_4)_2SO_4$
	0.20	KH_2PO_4
	0.03	$CaCl_2$. 2(H_2O)
	0.03	$MgSO_4$. 7(H_2O)
	0.03	Urea
	0.1	Peptone (casein)
	0.0005	$FeSO_4$. 7(H_2O)
	0.0016	$MnSO_4$. H_2O
	0.0014	$ZnSO_4$. 7(H_2O)
	0.0002	$CoCl_2$. 2(H_2O)
	1.00	D(+)-Glucose monohydrate
**DPY**	2.00	Dextrin from potato starch
	1.00	Polypeptone
	0.5	Yeast extract
	0.5	KH_2PO_4
	0.05	$MgSO_4$. 6(H_2O)
**DPY + 1 % glucose**	2.00	Dextrin from potato starch
	1.00	Polypeptone
	0.5	Yeast extract
	0.5	KH_2PO_4
	0.05	$MgSO_4$. 6(H_2O)
	1.00	D(+)-Glucose Monohydrate
**SS**	5.00	Soya sauce
	5.00	Sucrose
**PDB**	2.40	Potato Dextrose broth
**CM**	2.00	Cottonseed flour
	10.00	Lactose monohydrate
**CMP**	3.50	Czapex Dox broth
	2.00	Maltose monohydrate
	1.00	Polypeptone

Table S1.2: Solid media:

Agar	Composition [% (*w/v*)]	Ingredients
**SM-URA**	0.17	Yeast nitrogen base
	0.50	$(NH_4)_2SO_4$
	2.00	D(+)-Glucose Monohydrate
	0.077	Complete supplement mixture minus Uracil
	2.50	Agar
**LB agar**	0.5	Yeast extract
	1.00	Tryptone
	0.5	NaCl
	1.5	Agar
**ME agar**	1.28	Malt extract
	0.08	Peptone from Soya
	0.24	Glycerol
	0.28	Dextrin from potato starch
	1.5	Agar
**ME(BF) agar**	2.00	Malt extract
	0.10	Peptone ex Soya
	2.00	D(+)-Glucose monohydrate
	1.50	Agar
**YPAD agar**	1.00	Yeast extract
	2.00	Tryptone
	2.00	D(+)-Glucose Monohydrate
	0.03	Adenine
	1.50	Agar
**Mandel's Agar without Peptone, withGlycerol, with Glucose**	0.03	Urea
	0.14	$(NH_4)_2SO_4$
	0.02	KH_2PO_4
	0.03	$CaCl_2$. 2(H_2O)
	0.03	$MgSO_4$. 6(H_2O)
	0.01	Tween 80
	1.00	Carboxymethyl cellulose
	0.0005	$FeSO_4$. 7(H_2O)
	0.0016	$MnCl_2$. 4(H_2O)
	0.0017	$ZnCl_2$
	0.0002	$CoCl_2$. 6(H_2O)
	1.00	Glycerol
	1.00	D(+)-Glucose Monohydrate
	1.5	Agar
**Mandel's Agar without Peptone, with Glycerol, with Glucose, with Sorbitol**	0.03	Urea
	0.14	$(NH_4)_2SO_4$
	0.02	KH_2PO_4
	0.03	$CaCl_2$. 2(H_2O)
	0.03	$MgSO_4$. 6(H_2O)
	0.01	Tween 80
	1.00	Carboxymethyl cellulose
	0.0005	$FeSO_4$. 7(H_2O)
	0.0016	$MnCl_2$. 4(H_2O)
	0.0017	$ZnCl_2$
	0.0002	$CoCl_2$. 6(H_2O)
	1.00	Glycerol
	1.00	D(+)-Glucose Monohydrate
	18.22	Sorbitol (1M)
	1.5	Agar
**DPY agar**	2.00	Dextrin from potato starch
	1.00	Polypeptone
	0.5	Yeast extract
	0.5	KH_2PO_4
	0.05	$MgSO_4$. 6(H_2O)
	2.50	Agar
**PD agar**	2.40	Potato Dextrose broth
	1.50	Agar

**DPY + 1 % glucose agar**	2.00	Dextrin from potato starch
	1.00	Polypeptone
	0.5	Yeast extract
	0.5	KH_2PO_4
	0.05	$MgSO_4$. 6(H_2O)
	1.00	D(+)-Glucose monohydrate
	2.50	Agar

Table S1.3: Solutions:

Solutions	Components
**Protoplasting solution for *T. reesei***	1.2 M Sorbitol, 100 mM KH_2PO_4, pH 5.6
**Washing solution for *T. reesei***	1.2 M Sorbitol, 10 mM Tris-HCl, pH 7.5
**Resuspending solution for *T. reesei***	1 M Sorbitol, 10 mM $CaCl_2$, 10 mM Tris-HCl pH 7.5
**PEG 6000 solution for *T. reesei***	50 mM $CaCl_{2,}$ 10 mM Tris-HCl pH 7.5, 25 % (*w/v*) PEG6000, pH 7.5
**10 x TE buffer**	100 mM Tris HCl pH 8, 10 mM EDTA
**Loading-dye (6 x)**	0.25 % (*w/v*) bromophenol blue, 30 % (*v/v*) glycerine, 0.25 % (*w/v*) xylene cyanol
**50 X TAE buffer**	2 M Tris acetate, 0.05 M EDTA, pH 8.3

Table S1.4: Enzymes:

Name	Function	Reference
***Asc*I**	Restriction enzyme	NEB
***Pac*I**	Restriction enzyme	NEB
***Swa*I**	Restriction enzyme	NEB
***Not*I**	Restriction enzyme	NEB
**Gateway™ LR Clonase™ II Enzyme Mix**	contains integrase, excisionase and integration host factor	Thermo Fisher Scientific
**OneTaq® 2 X Master Mix with Standard Buffer**	DNA polymerase with agarose gel running buffer	NEB
**Proteinase K**	Proteinase	Thermo Fisher Scientific
**Q5® High-Fidelity DNA Polymerase 2 x Master Mix**	DNA polymerase with enhanced proofreading ability	NEB
***Trichoderma harzianum* lysing enzyme**	Cell Dissociation and cell lysis; preparation of protoplasts	Sigma
Driselase	Cell Dissociation and cell lysis; preparation of protoplasts	Sigma

Table S1.5: Antibiotics:

Name	Stock concentration / mg/mL in H_2O	Final concentration / µg/mL
**Carbenicillin disodium salt (Carb)**	50	50
**Kanamycin (Kan)**	50	50
**Hygromycin B (hyg)**	50	100-150

1.1. Preparation of Waste Media

For potato, orange, banana and kiwi peels, 50 g of the peels were cut into small pieces and autoclaved with 100 ml pure water at 121 °C for 15 min. For coffee press, 10 g coffee press leftover from cofffee machine was autoclaved (121 °C/ 15 min) with 100 ml pure water. For barley straw, 5 g barely straw (donated from a horse stable near Hannover, Germany) were cut into small pieces and autoclaved with 100 ml pure water at 121 °C for 15 min.

2. Bacterial and Fungal Strains

Table S2.1: Bacterial and fungal strains:

Strain	Genotype	Origin	Ref
E. coli Top10	F-*mcrA* Δ(*mrr-hsd*RMS-*mcr*BC) Φ80*lac*ZΔM15 Δ*lac*X74 *rec*A1 *ara*D139 Δ(*araleu*)7697 *gal*U *gal*K *rps*L(Str^R) *end*A1 *nup*G	Thermo Fisher Scientific	-
E. coli ccdB survival cells	F-*mcrA* Δ(*mrr-hsd*RMS-*mcr*BC) Φ80*lac*ZΔM15 Δ*lac*X74 *rec*A1 *ara*Δ139 Δ(*ara-leu*)7697 *gal*U *gal*K *rps*L (Str^R) *end*A1 *nup*G *fhu*A::IS2	Thermo Fisher Scientific	-
S. cerevisiae CEN.PK2	*MATa/α URA3-52/URA3-52 trp1-289/trp1-289 leu2-3_112/leu2-3_112 his3 Δ1/his3 Δ1MAL2-8C/MAL2-8C SUC2/SUC2*	Hahn group, Hannover	[1]
T. reesei QM6a Δtmus53	*Δtmus53*	Mach-Aigner group, Austria	[2]
T. reesei QM6a Δtmus53 Δpyr4	*Δtmus53 Δpyr4*	Mach-Aigner group, Austria	[2]

3. Vectors and Oligonucleotides

Table S3.1. Vectors used in this work:

Name	Origin	Ref
**pTY·*argB***	Lazarus group, Bristol	[3]
**pEYA·*aspks1***	Dr Raissa Shor	[4]
**pEYA·*tenS***	Cox group	[5]
**pTYGS·*argB·tenS·tenC***	Cox group	[5]

Table S3.3 Primers and oligonucleotides:

NO.	Name	sequence (5'-3')	Note
**11**	ITS1	TCCGTAGGTGAACCTGCGG	ITS sequencing
**12**	ITS4	TCCTCCGCTTATTGATATGC	ITS sequencing
**91**	PgdpA plugF	CTTTTCTTTTCTCTTTCTTTTCCCATCTTC	Amplify PgpdA patch
**92**	PgpdA plugR	TGACCTCCTAAAACCCCAGTG	Amplify PgpdA patch
**682**	TenS·TDS-F	CAAGCTCAGTCACTCGCTCA	Check TenS insertion
**683**	TenS·TDS-R	CACTATCCTCGCCGATGCTT	Check TenS insertion
**1327**	Hyg LF RP	CGTCAGGACATTGTTGGAG	SorBC KO left fragment (Bipartite method)
**1328**	Hyg RF FP	GCTTTCAGCTTCGATGTAGG	SorBC KO right fragment (Bipartite method)
**1663**	Aspks1-P1-F	CGCATTACTCCCATCATCAC	*aspks1* sequencing primer
**1664**	aspks1-seq-P2	CGCCAACTGCAAAAAGAAGG	*aspks1* sequencing primer
**1665**	aspks1-seq-P3	CACAGCAACCGGAAACCTCC	*aspks1* sequencing primer
**1666**	aspks1-seq-P4	CAGCCGGGATCAAGTTTCCT	*aspks1* sequencing primer
**1667**	aspks1-seq-P5	ACACGCTTTCCACTCGGTTC	*aspks1* sequencing primer
**1668**	aspks1-seq-P6	CAACGTTAGTGGACAATGGA	*aspks1* sequencing primer
**1669**	aspks1-seq-P7F	AGCAAGTTGGTACAATGGAG	*aspks1* sequencing primer
**1670**	aspks1-seq-P7R	GCTTCGAGCCAGTTTTGCTA	*aspks1* sequencing primer
**1671**	aspks1-seq-P8	ACTAGCCTCGAACGAGTGCG	*aspks1* sequencing primer
**1672**	aspks1-seq-P9	ACTGGGACAGATCGCAGGCA	*aspks1* sequencing primer
**1673**	aspks1-seq-P10R	TCACAACAAGAACCCCACCT	*aspks1* sequencing primer
**1678**	SorBC-KO-long-RF	GCCAACTTTGTACAAAAAAGCAGGCTCCGCATGGCGGCCTCAAGTACACG	SorBC KO cassette construction
**1679**	SorBC-KO-long-RR	ACGTATTTCAGTGTCGAAAGATCCACTAGAATGGCTCAGACTTGCCTTGG	SorBC KO cassette construction
**1680**	SorBC-KO-long-HygF	ATCAAACGCGCCAAGGCAAGTCTGAGCCATTCTAGTGGATCTTTCGACAC	SorBC KO cassette construction
**1681**	SorBC-KO-long-HygR	CAACATCCGCCTTCGCAGTTGACCCATCGCCAGGTCGAGTGGAGATGTGG	SorBC KO cassette construction
**1682**	SorBC-KO-long-LF	AGCGCCCACTCCACATCTCCACTCGACCTGGCGATGGGTCAACTGCGAAG	SorBC KO cassette construction
**1683**	SorBC-KO-long-LR	AACTTTGTACAAGAAAGCTGGGTCGGCGCGCTAATGCTTCTCTAACACCT	SorBC KO cassette construction
**1684**	PF-Pyr4-5'flank	AAGAAAGAAGTAAAGAAAGGCATTTAGCAAAGATCTCGAGATAGTATCTC	pTYGS-*pyr4*-P_{pdc} vector construction
**1685**	PR-Pyr4-5'flank	GACAAGAGCTCTTTCTCAGCTCTTAATTAATCCGGAGTAGCTCTTCACTG	pTYGS-*pyr4*-P_{pdc} vector construction
**1686**	PF-pyr4-3' flank	ACCAGTGAAGAGCTACTCCGGATTAATTAAGAGCTGAGAAAGAGCTCTTG	pTYGS-*pyr4*-P_{pdc} vector construction
**1687**	PR-pyr4-3' flank	TCTGCCGCGCCAAGTAGCCCTGGAAGTCCTCGTGCGTCTCGTTGTGCTCG	pTYGS-*pyr4*-P_{pdc} vector construction
**1688**	PF-Ppdc	CACCGCCCGCGAGCACAACGAGACGCACGAGGACTTCCAGGGCTACTTGG	pTYGS-*pyr4*-P_{pdc} vector construction
**1689**	PR-Ppdc	GCTTTTTTGTACAAACTTGTGATATCCAATGATTGTGCTGTAGCTGCGCT	pTYGS-*pyr4*-P_{pdc} vector construction
**1690**	Primer1- pyr4	GATGCCCTCGTTCTGTCCACAATTTCTTTCACGAAAAAGGTCTGACTGGG	pTYGS-*pyr4*-P_{pdc} vector construction
**1691**	Primer2- pyr4	ATTACTGAATGAGATACTATCTCGAGATCTTTGCTAAATGCCTTTCTTTA	pTYGS-*pyr4*-P_{pdc} vector construction
**1692**	AscI plug F	TGCCATCGAAGGAAGCAAAAGGTCAGGCTT	pTYGS-*pyr4*-P_{pdc}- P_{cDNA1}-P_{TReno} vector construction
**1693**	AscI plug R	CTTCGTCCGTCCTCGAAAGATGGAATTCAT	pTYGS-*pyr4*-P_{pdc}- P_{cDNA1}-P_{TReno} vector construction
**1694**	PcDNA1-PF	CGTGAACCATCACCCAAATCAAGTTTTTTATTTCCCCAGACAATGATGGT	pTYGS-*pyr4*-P_{pdc}- P_{cDNA1}-P_{TReno} vector construction

**1695**	PcDNA1-PR	TATGCGTTATGAACATGTTCCCATTTAAATTTGAGAGAAGTTGTTGGATT	pTYGS-*pyr4*-P_{pdc}- P_{cDNA1}-P_{TReno} vector construction
**1696**	TR-Peno-PF	GACCCACTGGGGTTTTAGGAGGTCAATTGTTTTGAAGCTATTTCAGGTGG	pTYGS-*pyr4*-P_{pdc}- P_{cDNA1}-P_{TReno} vector construction
**1697**	TR-Peno-PR	CTGGTAGACGTCATATAATCATATTTAAATTGATTCCGTCCTGGATTGCC	pTYGS-*pyr4*-P_{pdc}- P_{cDNA1}-P_{TReno} vector construction
**1698**	Plug-TReno PF	TCCATCTCGCCGAGAAGTGG	pTYGS-*pyr4*-P_{pdc}-*tenS*- P_{cDNA1}-*tenC*-P_{TReno} vector construction
**1699**	Plug-TReno-PR	TATAGCTACTAGCGACGGTC	pTYGS-*pyr4*-P_{pdc}-*tenS*- P_{cDNA1}-*tenC*-P_{TReno} vector construction
**1700**	TenC-PcDNA-PF	CTTTTTGATCAATCCAACAACTTCTCTCAAATGGCAGCCATCTCTTCCCC	pTYGS-*pyr4*-P_{pdc}-*tenS*- P_{cDNA1}-*tenC*-P_{TReno} vector construction
**1701**	TenC-PcDNA-PR	TTTCATTCTATGCGTTATGAACATGTTCCCTCAGGGCAGCGCCTCCTCTG	pTYGS-*pyr4*-P_{pdc}-*tenS*- P_{cDNA1}-*tenC*-P_{TReno} vector construction

4. Microbiological Methods.

4.1. *Escherischia coli*

Growth and Maintenance

E. coli strains were cultivated on liquid and solid LB medium with the apropriate antibiotic. Cultures were incubated at 37 °C and 200 rpm for 16-24 h. Glycerol stocks (25 % glycerol) were maintained at -80 °C for long term storage of the strains.

Transformation of Chemically Competent *E. coli*

Competent *E. coli* cells stored at -80 °C were thawed on ice for 5 min. 1-10 µL vector DNA were added and cells were stored on ice for 20-30 min. Heat shock was conducted at 42 °C for 30-45 s and cells were immediately placed on ice for 2 min. 250 µL SOC medium were then added and samples were incubated at 37 °C with 300 rpm shaking for 1 h. The cells were spun down for 15 s and 250 µL of the supernantatnt was discarded. The bacterial cells were then suspended in the remaining supernatant and spread on LB agar plates containing the appropriate antibiotic and incubated over night at 37 °C.

4.2. *Saccharomyces cerevisiae*

Growth and Maintenance

S. cerevisiae was cultivated on YPAD agar and incubated at 30 °C for three to five days. A single colony was used to inoculate 10 ml liquid YPAD media and was then grown overnight at 30 °C and 200 rpm. After transformation with URA3 containing plasmids, *S. cerevisiae* strains was grown on SM-URA agar plates at 30 °C for three to five days.

Preparation and transformation of competent *S. cerevisiae*

The high efficiency version of LiAc/SS carrier DNA/PEG protocol developed by Gietz and Schiestl [6] was used for the preparation and transformation of competent *S. cerevisiae*. A single colony was transferred into 10 mL YPAD medium and incubated over night at 30 °C with 200 rpm shaking. This starter culture was added to 40 mL YPAD medium in a 250 mL Erlenmeyer flask and incubated for another 4.5 h at 30 °C with 200 rpm shaking. Culture was harvested by centrifuging at 3000 x g for 5 min. The cell pellet was first washed with 25 mL dd.H_2O before it was resuspended in 1 ml dd.H_2O, transferred into a 1.5 mL reaction tube and centrifuged at 13000 x g for 30 s and supernatant discarded. The pellet was suspended in 400 µL water and each 100 µL were transferred into a separate 1.5 mL reaction tube.

For yeast recombination based cloning using *S. cerevisiae*, 100 µL aliquots of yeast competent cells were centrifuged for 30 s at 13000 x g and the supernatant was discarded. The following components were added to the pellet in order: 240 µL PEG solution (50 % (*w/v*) polyethylene glycol 3350), 36 µL LiAc (1 M), 50 µL denaturated salmon testis DNA (2 $mg.mL^{-1}$), 34 µL DNA containing the linearized plasmid and desired inserts obtained by PCR in equimolar concentration. The PCR fragments contain each 30 bp overlap at both 5` and 3` with the cut sites of the vector fragments to facilitate homologous recombination. The uncut plasmid was used for positive and linearized plasmid or dd.H_2O was used for negative control. The pellet was resuspended in the transformation mix by vigorous vortexing and incubated for 40 min at 42 °C. Cells were pelleted by centrifugation at 13000 xg for 30 s and supernatant was removed. The pellet was resuspended in 500 µL dd.H_2O and each 250 µL were spread on selective SM-URA plates, which were incubated for three to four days at 30 °C. Yeast plasmid was extracted using a Zymoprep™ Yeast Plasmid Miniprep II kit (Zymo reserach, Orange, California, USA).

4.3. *Trichoderma reesei*

Growth and Maintenance

T. reesei was grown on ME or PD agar at 28 °C. Transformed *T. reesei* strains were grown either on minimal media (Mandel`s agar without peptone + 1 % glucose + 1 % glycerol) or DPY+ 1 % glucose agar and incubated at 28 °C for 2-7 days. For glycerol stocks 5 mL of dd.H_2O were added to the plate and spores were scraped off using a sterile spatula. 800 µL of spore solution were supplemented with 800 µL glycerol (50 %) and stored in a cryovial at -80 °C. For liquid cultures 100 mL DPY + 1 % glucose medium or ME (BF) media was inoculated with *T. reesei* spore solution and incubated at 28 °C for three to six days with 110 rpm shaking.

PEG-Mediated Transformation of *Trichoderma reesei*

Protoplast preparation and *T. reesei* transformation was done following a modified protocol from that published by Gruber *et al* [7] The conidia from sporulating plate or spores stored as glycerol stocks were inoculated in 100 ml PDB or DPY media and incubated at 28 °C with shaking at 110 rpm for 2 days. The germinated conidia were separated from the liquid medium by filtration through sterile Miracloth. 20 ml of filter-sterilized protoplasting solution containing 12 mg/ml *Trichoderma* lysing enzyme or 10 mg/ml Driselase™ enzyme was used to resuspend the mycelia. The mixture was then incubated at 28 °C with shaking at 110 rpm for 2-3 hours. Protoplasts were separated from undigested mycelial debris by filtering through Miracloth and then centrifugation at 3500 x g for 3 minutes to separate protoplasts. Additional washing step (optional) was done by suspending the protoplasts in 15 ml washing solution for *T. reesei* then centrifugation at 3500 x g for 3 min. Protoplasts were then resuspended in 300 µl resuspension solution.

The concentration of the protoplasts was assessed microscopically. 10 µL of plasmid DNA was mixed with 100 µl of the protoplast suspension and 200 µl of PEG 6000 solution and the mixture was incubated on ice for 20 min. 0.5 ml of PEG 6000 solution and 1 ml resuspension solution were added to the transformation mixture and then the whole mixture was incubated at room temperature for 5 min. Aliquots of 300 ml of the solution were mixed with 20 ml of 50 °C Mandel's Agar without peptone and with 1 % glycerol, 1 % glucose and 1 M sorbitol. The plates were incubated at 28 °C for 3-7 days until the colonies appeared. For selection on uridine deficient media, the selection was repeated three times by picking the colonies using sterile tooth picks and transferring them into new minimal media plates (Mandel's Agar without peptone and with 1 % glycerol and 1 % glucose).

5. Molecular Biology Methods

All enzymes used was purchased from NEB and Thermo Fisher scientific and was used according to the manufacturer's instructions using the supplied buffers.

5.1. Plasmid DNA Extraction from *E. coli*

Plasmid DNA was generally extracted from overnight culture of *E. coli* using the NucleoSpin® Plasmid Kit (Machery-Nagel), according to the manufacture's protocol. The plasmid DNA concentration of each isolation was measured using a Nanodrop spectrophotometer.

5.2. Plasmid DNA Extraction from *S. cerevisiae*

Plasmid DNA was isolated from the 2-3 days culture of *S. cerevisiae* using Zymoprep™ Yeast Plasmid Miniprep II Kit (Zymo Research, USA), according to the manufacture's protocol. All the colonies grown on one SM-URA agar plate after the yeast recombination were collected with a tooth pick and dissolved in 200 µl of solution 1 of the yeast miniprep kit. Then yeast DNA was isolated according to manufacturers' instructions and 10 µl dd.H_2O was used for the final elution. Generally, 3-10 µl of the isolated plasmid was transformed immediately to competent *E. coli* cells.

5.3. Fungal Genomic DNA

Fungal genomic DNA was isolated from about 100 mg of mycelia using the GeneElute™ Plant Genomic DNA Miniprep Kit (Sigma-Aldrich). All the extractions and reactions were done according to the manufacture's protocols.

5.4. Polymerase Chain Reaction (PCR)

PCR was used to amplify DNA fragments for both screening and cloning purposes. OneTaq® 2× Master Mix (NEB) was used for screening purposes, while Q5® High–Fidelity 2× Master Mix (NEB) was used for precise fragment amplification for cloning purposes. Both enzymes were used according to the manufacture's protocols.

5.5. Cloning Procedure

Restriction digest

All of restriction enzymes were purchased from NEB. The enzymatic reactions were conducted according to restriction digest protocols. The digested plasmids were then evaluated by agarose gel electrophoresis.

Gateway Cloning

Invitrogen Gateway® LR Clonase II enzyme mix was used to perform *in vitro* recombination between pE-YA entry plasmids and pTYGS expression plasmids. The enzyme was used according to the manufacturer's protocol with reduced reaction scale (reduced to half).

5.6. DNA Sequencing

DNA samples were sequenced by Eurofins Genomics (Ebersberg). 2 µL of 10 µM primer solution was added to templates consisting of at least 15 µg of purified DNA. The mixture was then sent for sequencing.

5.7. Agarose Gel Electrophoresis

Agarose gel electrophoresis was used to analyse DNA fragments and Rota safe DNA stain was used to visualize the DNA fragments. Generally, 1 % agarose solution was prepared by dissolving 1 g Agarose in 100 mL of 0.5 x TAE buffer. DNA samples were mixed with 6 X DNA Loading Dye and loaded on 1 % (*w*/*v*) agarose gels. 1 kb DNA Ladder (NEB) was used as a molecular DNA size

marker. Electrophoresis was carried out in horizontal gel tanks (BioRad) at 90 – 110 V for 20 - 35 min using 0.5 xTAE as running buffer. DNA was visualised under UV (254 nm) and photographed using Gel Doc™ XR+ Gel Documentation System (BioRad).

5.8. DNA recovery from PCR

NucleoSpin® Gel and PCR Clean–up Kit was used to directly purify PCR mixture according to the manufacture's protocol. The isolated DNA fragments were then evaluated by agarose gel electrophoresis.

5.9. *E. coli* colony PCR

To screen positive colonies of *E. coli*, part of each single colony was picked from the LB agar plate using a toothpick and dipped into 10 µl TE buffer in PCR tube. The cells were then disrupted by heating to 80 °C for 15 minutes followed by 55 °C for another 15 min using a thermocycler. Then 1 µl of the cell suspension was used for PCR (using OneTaq® 2× Master Mix, NEB), according to the manufacturers protocol.

6. Chemical Analysis

6.1. Solvents and Chemicals

All the chemicals and materials were purchased from one of the following companies: Bio-Rad (München), New England Biolabs (Beverly, MA, USA), Roth (Karlsruhe), Sigma Aldrich (Steinheim), and Thermo Fisher Scientific (Waltham, MA, USA).

6.2. Liquid Chromatography Mass Spectrometry (LCMS)

Analytical LCMS

Analytical LCMS data of the organic extracts were obtained using a Waters LCMS system that consisted of a Waters 2767 autosampler, a Waters 2545 pump and Phenomenex Kinetex column (2.6 µm, C_{18}, 100 Å, 4.6 x 100 mm) equipped with a Phenomenex Security Guard precolumn (Luna, C_5, 300 Å). Detection was performed by a diode array detector from 210 - 600 nm (DAD; Waters 2998 or Waters 996), an evaporative light scattering detector (ELSD; Waters 2424) and together with a mass spectrometry, Waters SQD-2 mass detector, operating simultaneously in ES^+ and ES^- modes between 150 and 1000 *m*/*z*. A solvent gradient was run over 15 min starting at 10 % acetonitrile/ 90 % HPLC grade water (+ 0.05 % formic acid) and ramping to 90 % acetonitrile. Flow rate was 1 mL/min and 20 µL of the sample was injected. Data were displayed using the software MassLynx.

Table S6.2. Program used for analytical LCMS:

Time/min	Water:acetonitrile / %
0-1	90:10
1-10	10:90
10-12	10:90
12-13	90:10
13-15	90:10

Preparative LCMS

Single compounds were purified from the raw organic extracts using a Waters mass–directed autopurification system. It compromises of a Waters 2767 autosampler, a Waters 2545 Pump and a Phenomenex Kinetex Axia column (5 µm, C_{18}, 100 Å, 21.2 x 250 mm) equipped with a Phenomenex Security Guard precolumn (Luna, C_5, 300 Å). A water/acetonitrile gradient depending on compound polarity was run over 15 minutes with a flowrate of 20 mL/min and a post-column flow split of 100:1. The minority flow was applied for simultaneous analysis by a diode array detector (Waters 2998) in the range 210 to 600 nm, an evaporative light scattering detector (ELSD; Waters 2424) and a Waters SQD-2 mass detector, operating in ES^+ and ES^- modes between 100 and 1000 *m*/*z*. Selected peaks were collected into test tubes and solvent was evaporated under reduced pressure.

6.3. High Resolution Mass Spectrometry (HRMS)

Compound was dissolved in methanol (1 mg/mL). High Resolution Mass Spectrometry was performed on a Q-Tof Premier mass spectrometer (Waters) coupled to an Acquity UPLC system (Waters). Electron spray Ionisation (ESI) mass spectroscopy was measured in positive or negative mode depending on the compound.

6.4. Nuclear Magnetic Resonance (NMR) analysis

Bruker Ascend 400 MHz, Bruker DRX 500 MHz or a Bruker Ascend 600 MHz Spectrometer (Bruker) were used for NMR measurements of the samples. Raw data were then analyzed using the software Bruker TopSpin 3.5. Chemical shifts are expressed in parts per million (ppm) in comparison to the Tetramethylsilane (TMS) standard and are referenced to the deuterated solvent.

6.5. Extraction of Fungal Cultures

For *T. reesei* transformants grown in 100 ml liquid media, around 100-200 mg of air-dried mycelia was collected by Büchner filtration and used for gDNA analysis if required. The remaining mycelia and media were homogenized using a hand blender and solids were removed from by Büchner filtration. The filtrate was acidified with 2 M HCL to pH 3-4 and extracted twice with ethyl acetate. The combined organic layers were dried over $MgSO_4$. The drying agent was removed by filatration and solvents were removed under vacuum. The resulting organic residue was dissolved in methanol or acetonitrile to a concentration of 5-10 mg/mL (analytical) or 50 mg/mL (preparative) and filtered over glass wool before LCMS analysis or purification.

6.6. Isolation of compounds from *T. reesei* transformants

Compounds were isolated from different *T. reesei* transformants using preparative LCMS (15 min gradient: 10 to 90 % water–acetonitrile). The NMR data of the isolated compounds is shown in ppm and compared to literature values.

3-methylorcinaldehyde 8 [8]

1 mg of compound **8** was purified from the extract of *T. reesei* QM6a *Δtmus53 ΔsorBC* P_{pdc2}*-aspks1* colony C. **HRMS** (ES^-) *m/z* calc. for $C_9H_9O_3$ $[M+H]^-$ 165.0552, found 165.0549. NMR, ^{1}H-NMR (500 MHz, CD_3OD), δ_H: 10.04 (1H, s, H-7), 6.23 (1H, s, H-5), 2.47 (3H, s, H-9) and 1.98 (3H, s, H-8).

3-methylorsellinic acid 9 [9]

15 mg of compound **9** was purified from the extract of *T. reesei* QM6a *Δtmus53 ΔsorBC* P_{pdc2}*-aspks1* colony C. **HRMS** (ES^-) *m/z* calc. for $C_9H_9O_4$ $[M+H]^-$ 181.0501, found 181.0502. NMR, ^{1}H-NMR (500 MHz, CD_3OD), δ_H: 6.20 (1H, s, H-5), 2.45 (3H, s, H-9) and 2.00 (3H, s, H-8). ^{13}C-NMR (125 MHz, CD_3OD), δ_C: 174.2 (*C*-7), 163.4 (*C*-2), 159.9 (*C*-4), 140.2 (*C*-6), 109.9 (*C*-5), 108.3 (*C*-3), 103.5 (*C*-1), 22.9 (*C*-9) and 6.5 (*C*-8).

7. Vector Construction.

Table S7.1: Vectors constructed in this work.

Vector	Vector Backbone	Oligonucleotides	Note
**pEYA *sorBC* Knockout**	pEYA	1678 + 1679	*sorB* flank
		1680 +1681	Hyg cassette
		1682 + 1683	*sorC* flank
**pTYGS·*pyr4*·P_{pdc}**	pTYGS·*argB*	1690 + 1691	*pyr4*
		1684 + 1685	*pyr*-5`flank
		1686 + 1687	*pyr4*-3`flank
		1688 + 1689	P_{pdc}
**pTYGS·*pyr4*·P_{pdc}·*aspks1***	pTYGS·*pyr4*·P_{pdc}	-------	LR with pEYA·*aspks1*
**pTYGS·*pyr4*·P_{pdc}·$P_{TRcDNA1}$·P_{TReno}**	pTYGS·*pyr4*·P_{pdc}	1694 + 1695	P_{cDNA1}
		1696 + 1697	P_{TReno}
		91 + 92	Patch P_{gpdA}
		1692 + 1693	Patch *Asc*I
**pTYGS·*pyr4*·P_{pdc}·*tenS***	pTYGS·*pyr4*·P_{pdc}·$P_{TRcDNA1}$·P_{TReno}	--------	LR with pEYA·*tenS*
**pTYGS·*pyr4*·P_{pdc}·*TenS*·$P_{TRcDNA1}$·*tenC***	pTYGS·*pyr4*·P_{pdc}·*tenS*	1700 + 1701	*tenC*
		1698 + 1699	Patch P_{TReno}

7.1. Construction of pEYA *sorBC* Knockout Vector

The pEYA backbone was linearised with *Asc*I. The vector pTHGS·eGFP [10] was used as a template to amplify the hygromycin resistance cassette using primers P1680 + P1681 (hph between P_{gpdA} and T_{trpC}). The gDNA of *T. reesei* QM6a Δ*tmus53* Δ*pyr4* was used to amplify ≈ 1 kb homologous arms to the *sorB* (P1678 + P1679) and *sorC* genes (P1682 + P1683). The linear vector and the three fragments (hygR casstte, *sorB* and *sorC* homologous arms) were recombined in yeast. The resulting plasmid was then confirmed using PCR with appropriate primers and partial sequencing and the correct plasmid was named, pEYA·sorBC-KO (Figure S7.1).

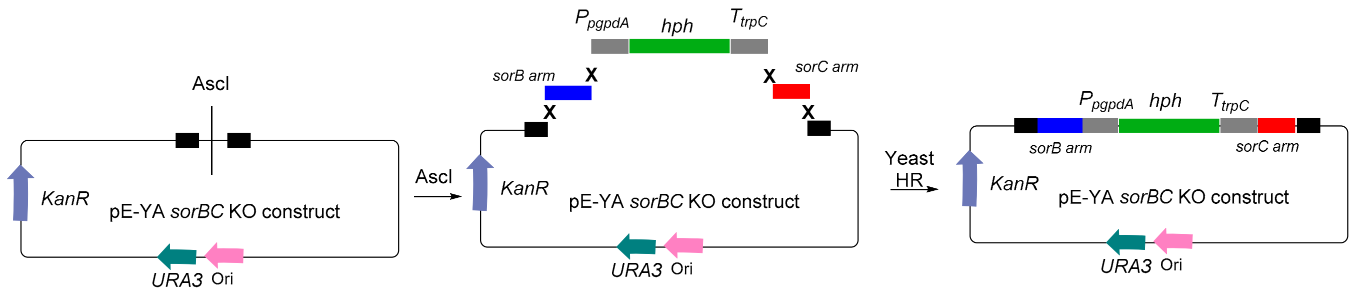

Figure S7.1. Plasmid construction to knockout the *sorB* and *sorC* genes in *T. reesei* by bipartite method.

7.2. Construction of pTYGS·*pyr4*·P_{pdc}

A vector was constructed for easy and fast integration of the genes in *T. reesei* QM6a·*Δtmus53*·*Δpyr4* gDNA. Four different PCR fragments were amplified with 30 bp flanking arms to each other and to the pTYGS·*argB* vector backbone. The *pyr4* selection marker was amplified from the gDNA of *T. reesei* QM6a *Δtmus53* using primers P1690 + P1691. Three different PCR fragments were amplified from the gDNA of *T. reesei* QM6a·*Δtmus53*·*Δpyr4* [2]: the native promoter P_{pdc} [11] was amplified using primers P1688 + P1689; the 3′-downstream region from the *pyr4* gene (*pyr4*-3′-flanking region) was amplified using primers P1686 + P1687; and the 5′-upstream region of the *pyr4* gene (*pyr4*-5′-flanking region) was amplified using primers P1684 + P1685. [2] The pTYGS·*argB* vector [3] was linearized using *Swa*I, then the linear vector and the four PCR fragments (*pyr4* selection marker , the native promoter P_{pdc}, *pyr4*-3′-

flanking region and *pyr4*-5′-flanking region) were recombined in yeast. The resulting plasmid was then confirmed using PCR with appropriate primers and partial sequencing and the correct plasmid was named pTYGS·*pyr4*·P_{pdc} (Figure S7.2).

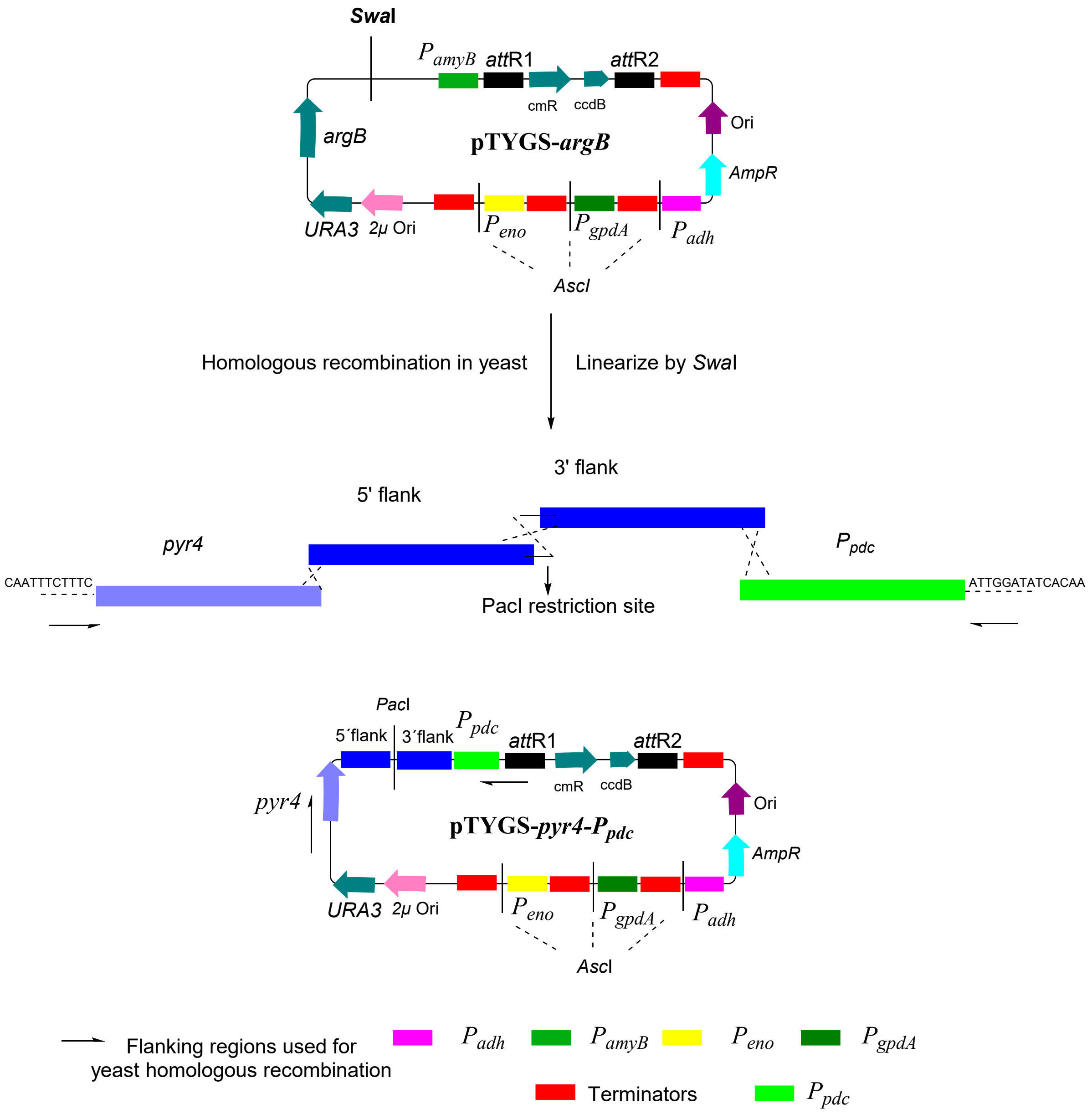

Figure S7.2. Construction of the vector with the *pdc* promoter (P_{pdc}) using yeast homologous recombination.

7.3. Construction of pTYGS·*pyr4*·P_{pdc}·*aspks1*

The constructed vector pTYGS·*pyr4*·P_{pdc} contains a Gateway® *in vitro* recombination cassette allowing insertion of any megasynth(et)ase gene present on a Gateway® entry vector. Therefore, the entry vector pEYA·*aspks1* [4] was combined with pTYGS·*pyr4*·P_{pdc} *in vitro* using components of the LR recombination kit (Thermofisher) and manufacturers procedures to insert *aspks1* in the cloning site downstream of P_{pdc} (Figure S7.3). The resulting vector, pTYGS·*pyr4*·P_{pdc}·*aspks1*, was confirmed by PCR and partial sequencing.

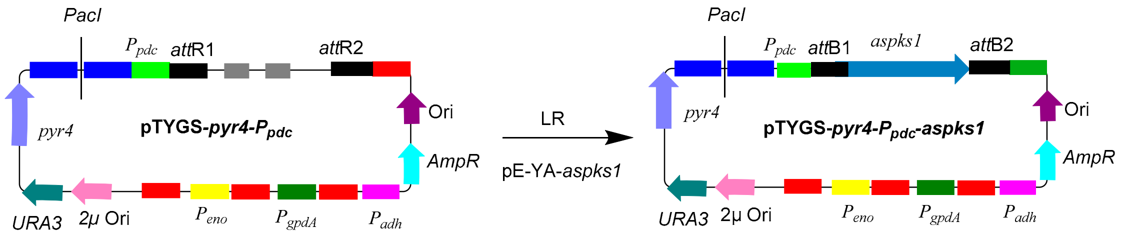

Figure S7.3. Construction of the vector pTYGS·*pyr4*·P_{pdc}·*aspks1* with *aspks1* under the activity of P_{pdc} using LR recombination with pEYA·*aspks1*.

7.4. Construction of pTYGS·*pyr4*·P_{pdc}·P_{cDNA1}·P_{TReno}

The two constitutive promoters, P_{cDNA1} and P_{TReno} were chosen to expand the vector pTYGS·*pyr4*·P_{pdc}. According to the published literature, P_{TReno} [11] is located from 102421 bp to 103910 bp on scaffold 4 and P_{cDNA1} [12,13] is located from 43726 bp to 44652 bp on scaffold 23 (Upstream of the *cDNA1* gene TRIREDRAFT_110879). The coding regions for the two native promoters P_{cDNA1} (P1694 + P1695) and P_{TReno} (P1696 + P1697) were amplified from gDNA of *T. reesei* QM6a *Δtmus53 Δpyr4*. The primers were designed in a way to insert a *Swa*I restriction site after each of the promoters, P_{cDNA1} and P_{TReno}, to facilitate later gene insertion by yeast homologous recombination. A patch for the P_{gpdA} promoter (P91 + P92) was amplified by PCR using pTYGS·*pyr4*·P_{pdc} vector as the template. Since the vector pTYGS-*pyr4*-P_{pdc} contained another site for *Asc*I restriction in the *pyr4*-3`-flanking region, a patch for this *Asc*I restriction site (P1692 + P1693) was also amplified by PCR using the vector pTYGS·*pyr4*·P_{pdc} as the template. The vector pTYGS-*pyr4*-P_{pdc} was linearized using *Asc*I then the linear vector and the four PCR fragments (The native promoter P_{cDNA1}, the native promoter P_{TReno}, P_{gpdA} promoter patch and the patch for the *Asc*I restriction site) were recombined in yeast. The resulting plasmid was then confirmed using PCR with appropriate primers and partial sequencing. The resultant vector was named pTYGS·*pyr4*·P_{pdc}·P_{cDNA1}·P_{Treno} (Figure S7.4).

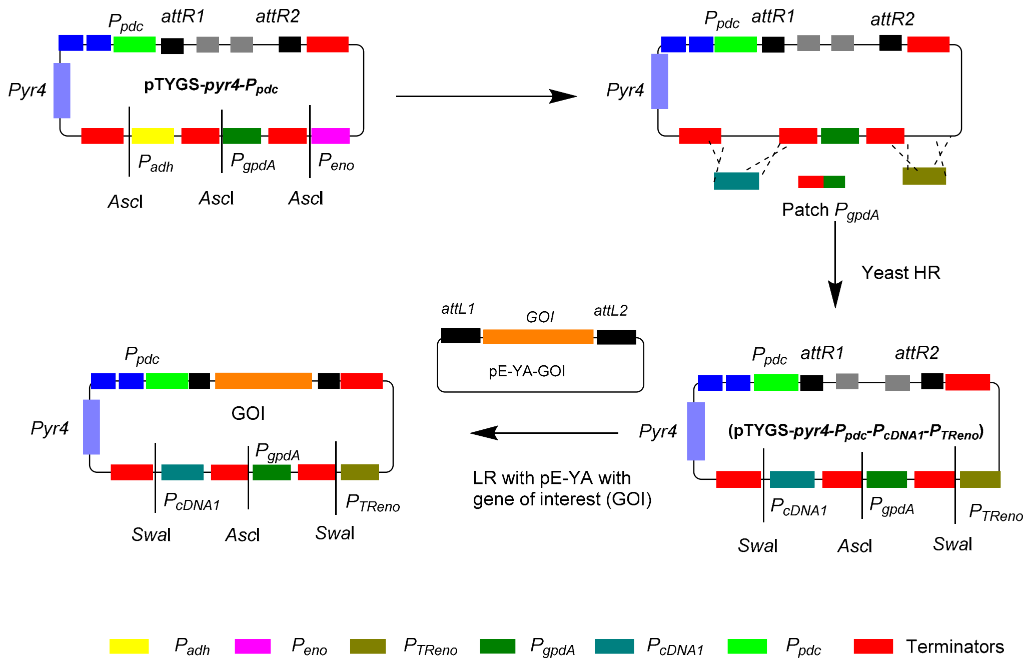

Figure S7.4. Construction of pTYGS·*pyr4*·P_{pd2}·P_{cDNA1}·P_{TReno} using yeast homologous recombination.

7.5. Construction of pTYGS·*pyr4*·P_{pdc}·*tenS*·P_{cDNA1}·*tenC*·P_{TReno}

The vectors containing *tenS* and *tenC* were available in the Cox group. [5] Gateway *in vitro* recombination between pTYGS·*pyr4*·P_{pdc}·P_{cDNA1}·P_{TReno} and pEYA·*tenS* [5] resulted in the construction of pTYGS·*pyr4*·P_{pdc}·*tenS*·P_{cDNA1}·P_{TReno}. The coding sequence for *tenC* was amplified from the vector pTYGS·*argB*·*tenS*·*tenC* [5] using primers with flanking regions to P_{cDNA1} and T_{adh} (P1700 + P1701). A patch for P_{TReno} was also amplified by PCR using pTYGS·*pyr4*·P_{pdc}·P_{cDNA1}·P_{TReno} as the template (P1698 + P1699). The vector pTYGS·*pyr4*·P_{pdc}·*tenS*·P_{cDNA1}·P_{Treno} was linearized by *Swa*I then the linear vector and the two PCR fragments (*tenC* gene and patch P_{TReno}) were recombined in yeast. The resulting plasmid was then confirmed using PCR with appropriate primers and partial sequencing and was named pTYGS·*pyr4*·P_{pdc}·*tenS*·P_{cDNA1}·*tenC*·P_{Treno} (Figure S7.5).

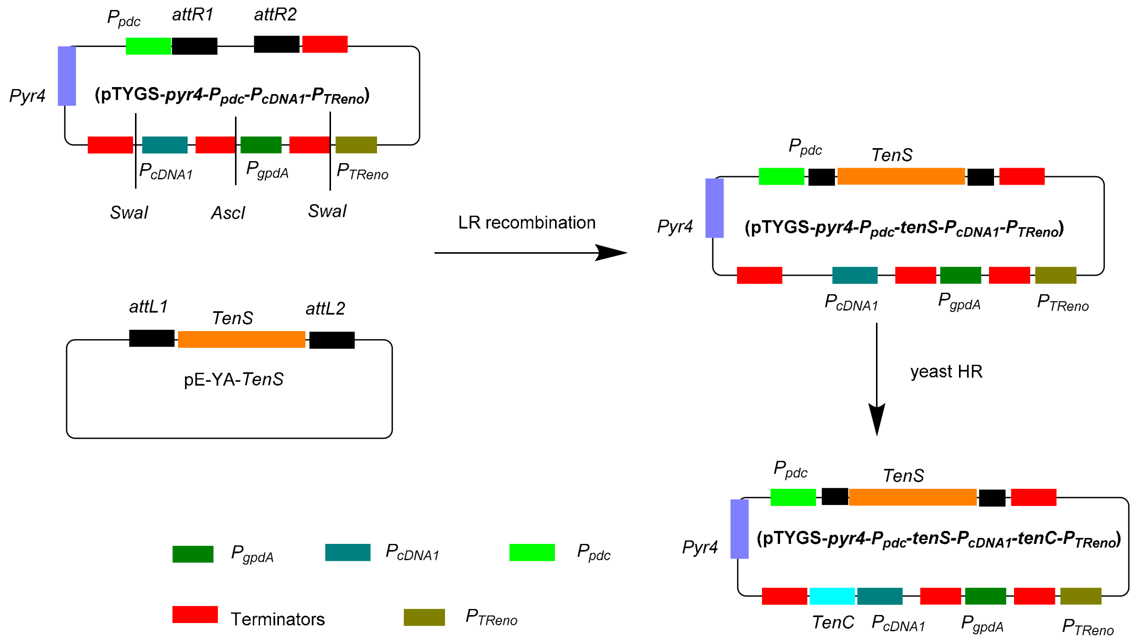

Figure S7.5. Construction of plasmid pTYGS·*pyr4*·P_{pdc}·*tenS*·P_{cDNA1}·*tenC*·P_{TReno} using LR and yeast homologous recombination.

8. Construction of a *T. reesei* QM6A·*Δtmus53·Δpyr4* Sorbicillin Deletion Strain.

8.1. Construction of bipartite deletion casette and transformation of *T. reesei* QM6a·*Δtmus53·Δpyr4*

The plasmid for the KO of *sorB* and *sorC* genes was constructed (Figure S7.1) then the plasmid was used as a PCR template to make two overlapping DNA fragtments (using primers 1327 + 1678 and 1328 + 1683) . These fragments were transformed into *T. reesei* using the bipartite method aiming to knock out the two key genes in the sorbicillin pathway, *sorB* and *sorC*. After three rounds of selection on PDA containing hygromycin (100 µg/ml), 18 hygromycin-resistant transformants were selected. Chemical analysis of six of them showed that one transformant, *T. reesei* QM6a·*Δtmus53·Δpyr4·ΔsorBC*, showed no production of any sorbicillin-related compounds compared to *T. reesei* QM6A·*Δtmus53·Δpyr4* after cultivation for seven days in DPY + 1 % glucose (Figure S8.1).

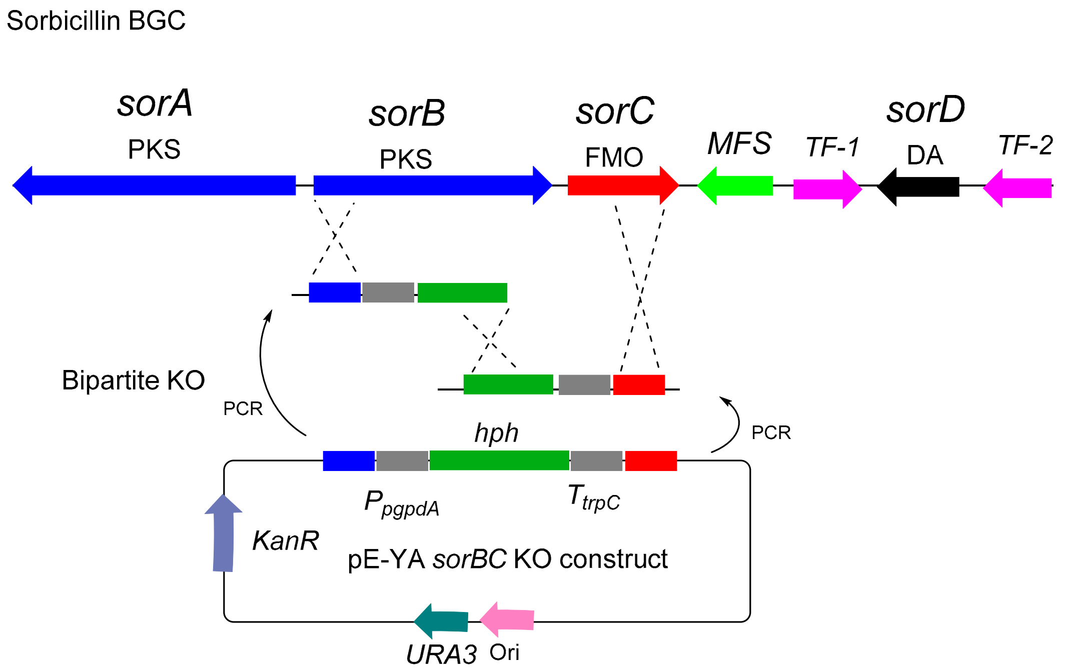

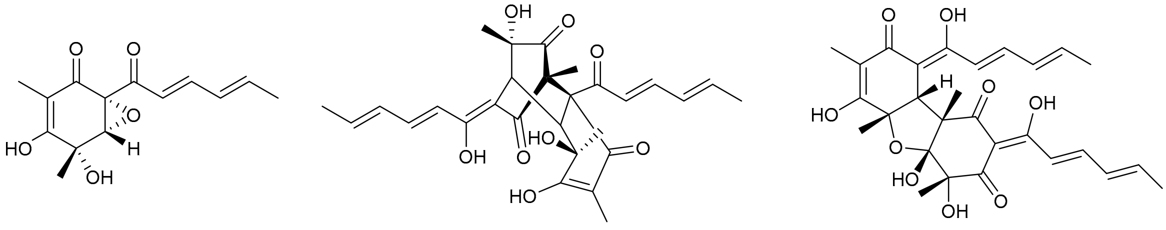

3
epoxysorbicillinol

4
bisorbicillinol

5
bisvertinolone

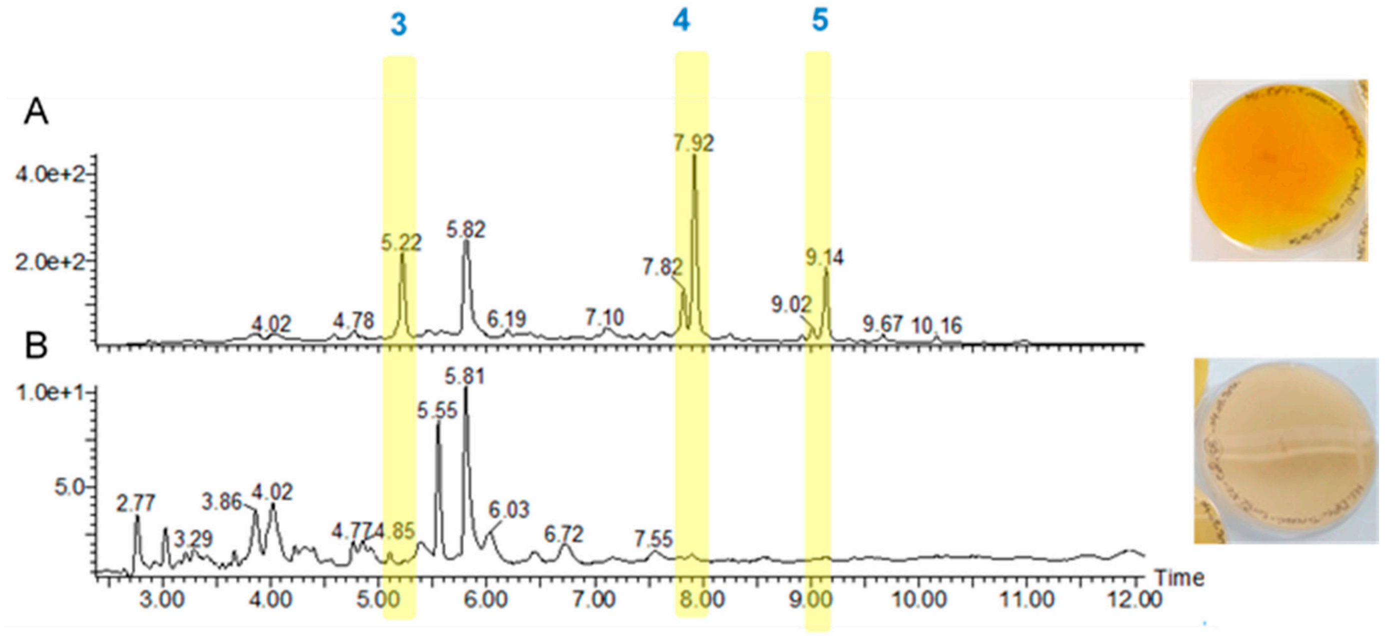

Figure S8.1 LCMS traces of the *sorBC*-KO transformants against *T. reesei* QM6a·*Δtmus53*·*Δpyr4* showing the absence of any sorbicillin-related compounds in only one out of the six transformants tested (*T. reesei* QM6a·*Δtmus53*·*Δpyr4*·*ΔsorBC*). **A**, WT; **B**, KO.

8.2. Analysis of *T. reesei* QM6a·*Δtmus53·Δpyr4·ΔsorBC*-5B in Different Media

PCR analysis of *T. reesei* QM6a·*Δtmus53·Δpyr4·ΔsorBC*-5B together with *T. reesei* QM6a·*Δtmus53·Δpyr4* using primers 1328 + 1683 showed the correct insertion of the knockout cassette in *T. reesei* QM6a·*Δtmus53·Δpyr4·ΔsorBC*-5B (Figure S8.2). ITS sequencing of *T. reesei* QM6a·*Δtmus53·Δpyr4·ΔsorBC*-5B confirmed that the strain indeed is *T. reesei*.

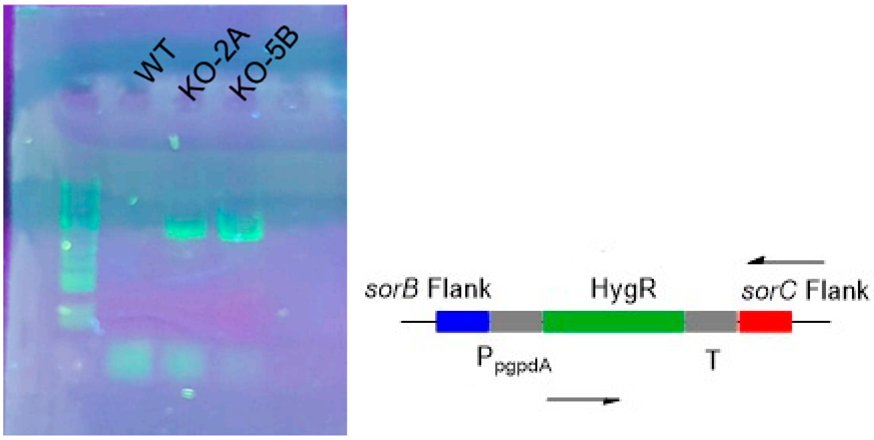

Figure S8.2. Genetic analysis of two different *T. reesei Δtmus53·Δpyr4·ΔsorBC* transformants (KO-2A and KO-5B) and the wild type *T. reesei Δtmus53·Δpyr4* showing the correct insertion of the KO cassette.

T. reesei QM6a·*Δtmus53·Δpyr4·ΔsorBC* was then cultivated in nine different media: PDB; ME (BF); DPY; DPY+1 % glucose; Mandel-Anderiotti + 1 % glucose; CM; CMP; Soya-soy-sucrose (SSS); and GNB for 7 days at 28 °C and 110 rpm (Figure S8.3). The new knockout strain showed no production of any sorbicillin related compounds under all the conditions tested. Although *T. reesei* QM6a·*Δtmus53·Δpyr4·ΔsorBC* did not produce any sorbicillin-related compounds, some new minor compounds with similar UV spectrum were produced (Figure S8.4).

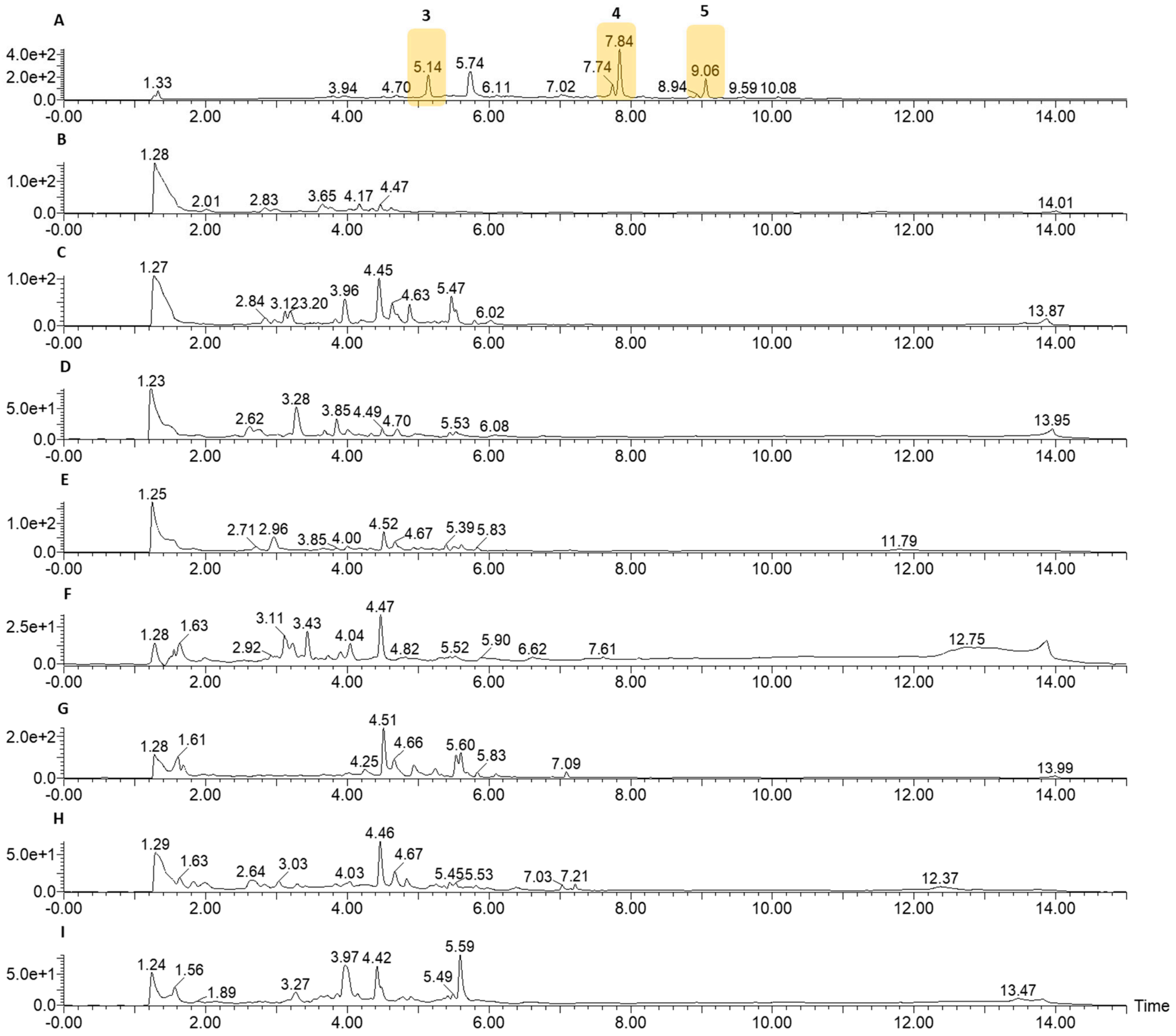

Figure S8.3. LCMS traces of *T. reesei* QM6a·*Δtmus53·Δpyr4·ΔsorBC* in different media showing no production of any sorbicillin-related compounds. **A**, DAD chromatograms of *T. reesei* QM6a *Δtmus53 Δpyr4* in PDB media; DAD chrmaotograms of *T. reesei* QM6a·*Δtmus53·Δpyr4·Δ*sorBC in different media: **B**, CM media; **C**, CMP; **D**, DPY; **E**, DPY+ 1 % glucose; **F**, GNB; **G**, ME(BF); **H**, SSS; **I**, PDB.

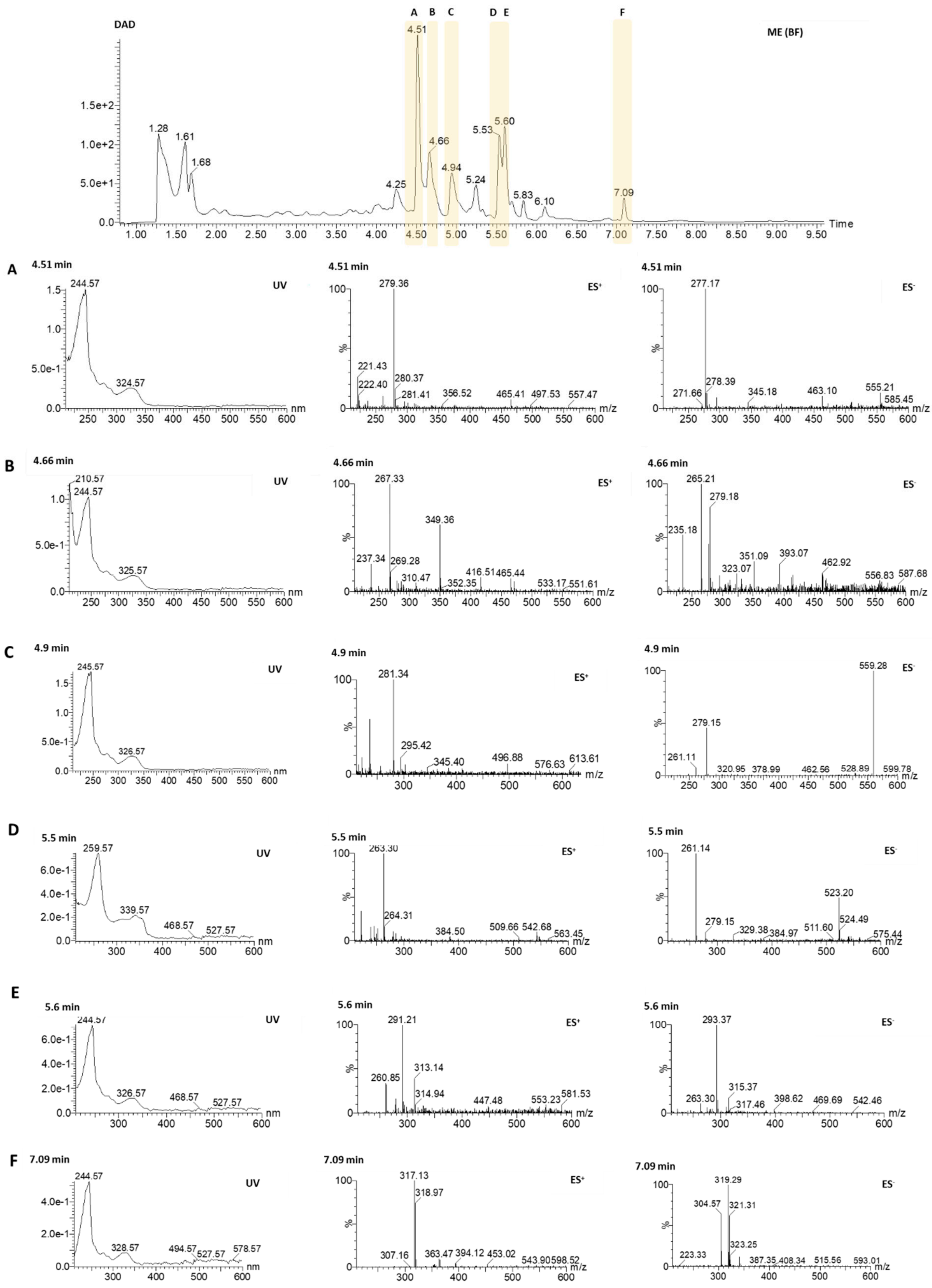

Figure S8.4. DAD chromatogram of *T. reesei* QM6a·*Δtmus53·Δpyr4·ΔsorBC* in ME (BF) media showing ES^+, ES^- and DAD spectrum for different compounds with similar UV spectrum produced by the strain. Compound A is citreoisocoumarin **7** (*m/z* 278); Compound C is likely to be the diol reduced form of **A** (*m/z* 280); **D** is likely a dehydrated form of **C** (*m/z* 262).

The compound eluting at 4.5 min (citreoisocoumarin **7**) was purified by mass-directed HPLC (3.5 mg from 1 L fermentation) and structure elucidation using NMR and comparison to published data (Table S8.1). HRMS analysis of the compound confirmed a molecular formula of $C_{14}H_{15}O_6$ $[M+H]^+$ (calc. 279.0869, found 279.0887). Citreoisocoumarin **7** and 6-*O*-methyl-citreoisocoumarin were previously reported from *Trichoderma* sp. HPQJ-34. [14]

Table S8.1. Chemical shifts of citroisocoumarin **7** in CD_3OD measured at 500 MHz (in comparison to literature data).

**Position**	**Found**		**Reported from *Trichoderma* sp. HPQJ-34** [14]		**Reported for (+) citreoisocoumarin** [15]	
	σ_H / *ppm*	σ_C / *ppm*	σ_H / *ppm*	σ_C / *ppm*	σ_H / *ppm*	σ_C / *ppm*
**1**		167.8		167.8		167.6
**3**		155.6		155.6		155.6
**4**	6.39, s	107.4	6.26, s	107.4	6.39, s	107.4
**4a**		141.1		141.1		141.1
**5**	6.32, s	103.8	6.34, s	102.7	6.32, s	103.8
**6**		167.4		167.3		167.2
**7**	6.32, s	102.7	6.26, s	99.9	6.32, s	102.7
**8**		164.9		164.8		164.8
**8a**		99.9		103.8		99.8
**9**	2.61 (dd, *J*= 14.5 and 8.0 Hz)<br>2.69 (dd)	41.9	2.55 (2H, dd, *J* = 14.3, 8.0 Hz)	41.9	2.63, m	41.9
**10**	4.45 (m)	66.4	4.40 (1H, dt, *J* = 12.1, 6.4 Hz)	66.4	4.45, m	66.4
**11**	2.69 (d, *J* = 6.3 Hz , 2H)	51.1	2.64 (2H, dd, *J* = 13.5, 5.4 Hz)	51.1	2.69, m	51.1
**12**		209.8		209.8		209.7
**13**	2.18, s	30.6	2.14, s	30.7	2.18, s	30.7

9. Expression of *aspks1* in *T. reesei* QM6a·*Δtmus53·Δpyr4·ΔsorBC*

9.1. Construction of QM6a·*Δtmus53·ΔsorBC·P_{pdc}·aspks1*

The vector pTYGS·*pyr4*·P_{pdc}·*aspks1* was linearized with *Pac*I and transformed into *T. reesei* QM6a·*Δtmus53·Δpyr4·ΔsorBC* using PEG-mediated transformation. After three rounds of selection on minimal media lacking uridine, eight transformants were obtained. Five transformants were cultivated on ME (BF) media for 6 days at 28 °C and 110 rpm. The cultures were extracted with ethyl acetate separately, evaporated under reduced pressure and submitted to LCMS. The chromatograms showed the production of 3-methylorsellinic acid **9** (RT 5.1 min, *m*/*z* 182 and 3-methylorcinaldehyde **8** (RT 5.9 min, *m*/*z* 166) after 6 days of growth (Figure S9.1).

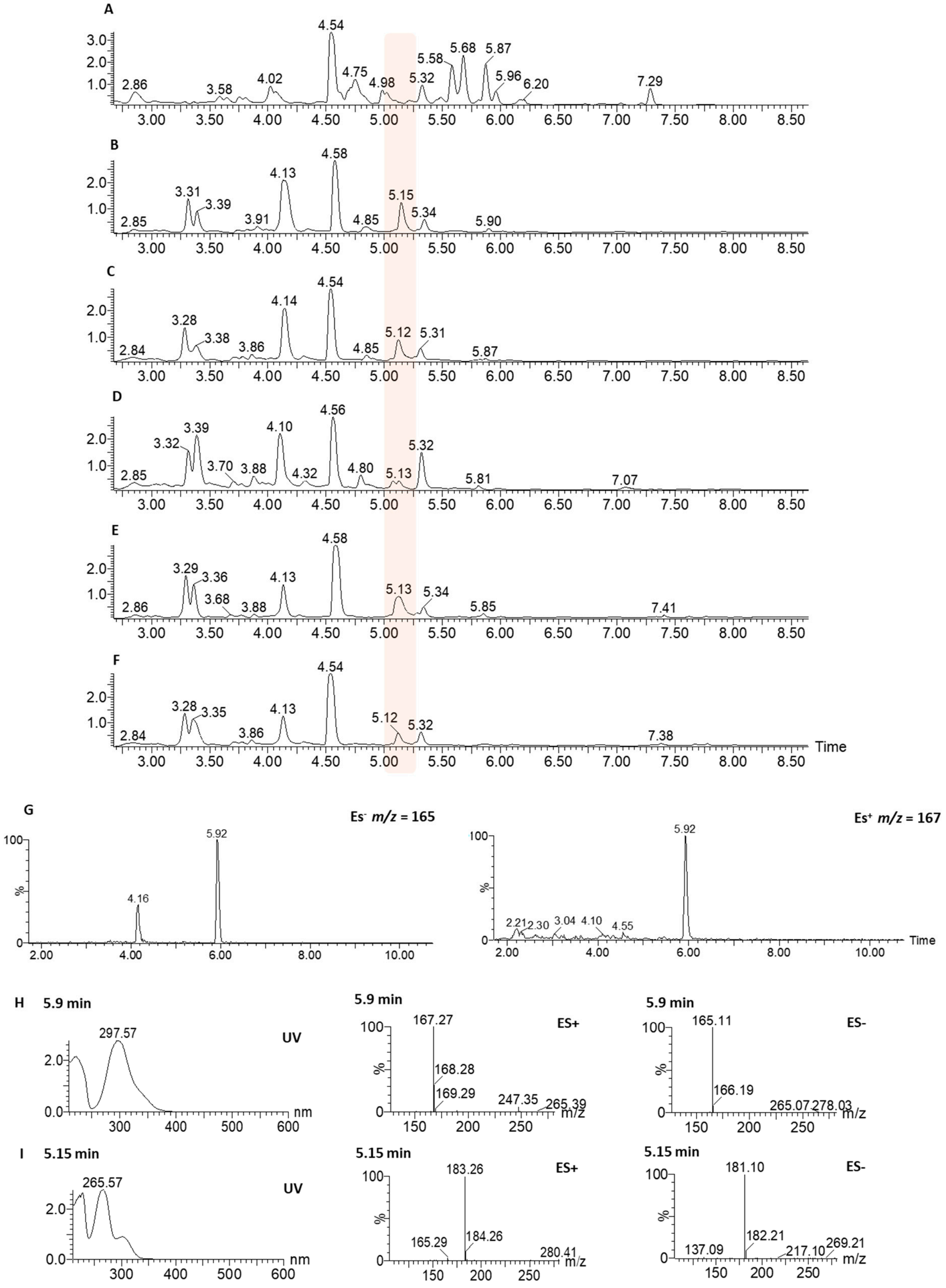

Figure S9.1. A, DAD LCMS traces of *T. reesei* QM6a·*Δtmus53*·*ΔsorBC* after six days in ME(BF) media; **B-F**, DAD LCMS traces of different *T. reesei* QM6a·*Δtmus53*·*ΔsorBC*·P_{pdc}·*aspks1* transformants after six days of cultivation in ME(BF) media showing the production of two new compounds in the *aspks1* transformants at T_R = 5.15 and 5.9 min. **G**, EIC of compound **8** at *m/z* 165 and 167; **H-I**, UV, ES+ and ES- data of the two new compounds **8** and **9**.

Three of the successful transformants were then cultivated in ME (BF) media for three days at 28 °C and 110 rpm. LCMS chromatograms of organic extracts of these cultures showed a prominent increase in the yield of 3-methylorcinaldehyde **8** in comparison to six-day cultures (Figure S9.2), with colony C (Figure S9.2-trace B) showing the best production levels.

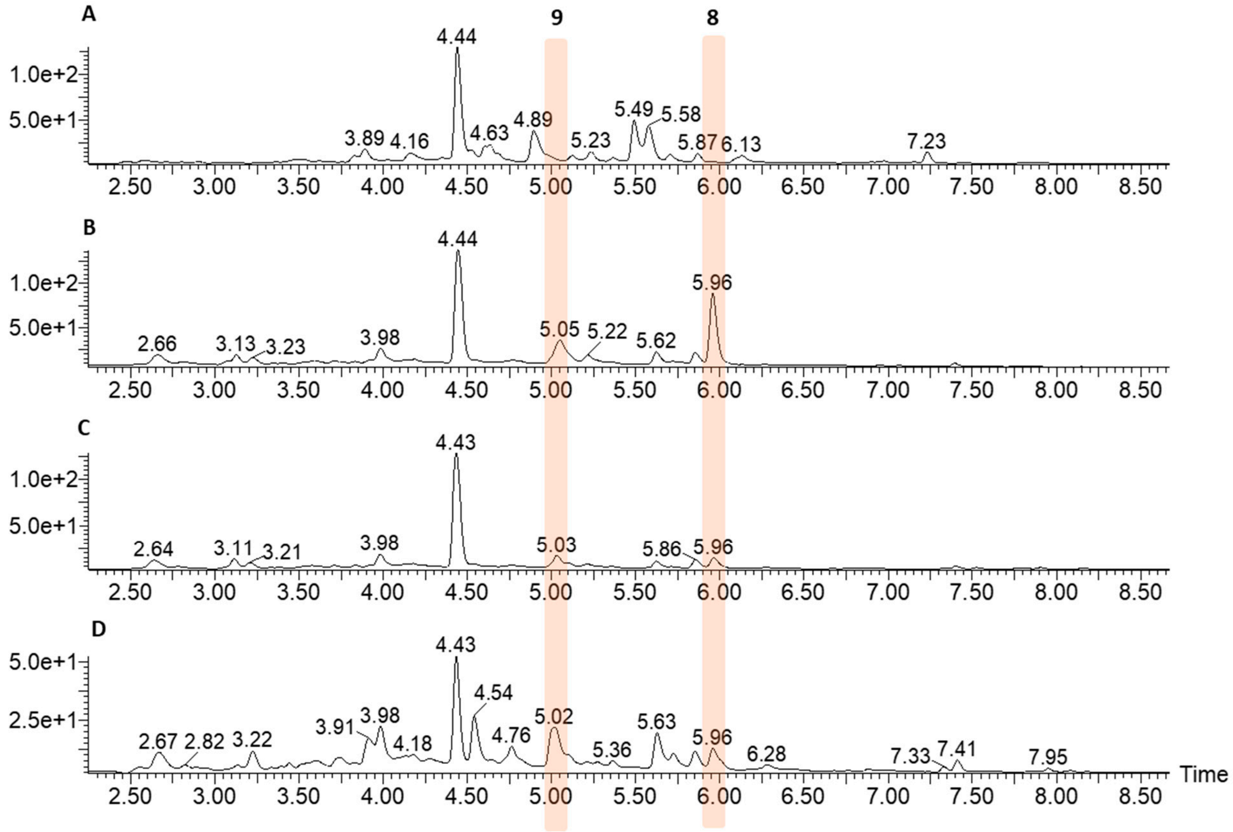

Figure S9.2. A, DAD LCMS traces of *T. reesei* QM6a·*Δtmus53·ΔsorBC* after three days in ME(BF) media; **B-D**, DAD LCMS traces of different *T. reesei* QM6a·*Δtmus53·ΔsorBC·*P_{pdc}*·aspks1* transformants after three days of cultivation in ME(BF) media showing the production of compounds **8** and **9**.

To establish the time course of the production of 3-methylorcinaldehyde **8** from *T. reesei* QM6a·*Δtmus53*·*ΔsorBC*·*P*$_{pdc}$·*aspks1* transformants, six different 500 ml baffled flasks each containing 100 ml DPY + 1 % glucose media were inoculated with this strain. The flasks were incubated at 28 °C and 110 rpm for one to six days and were extracted at 24 h intervals (Figure S9.3).

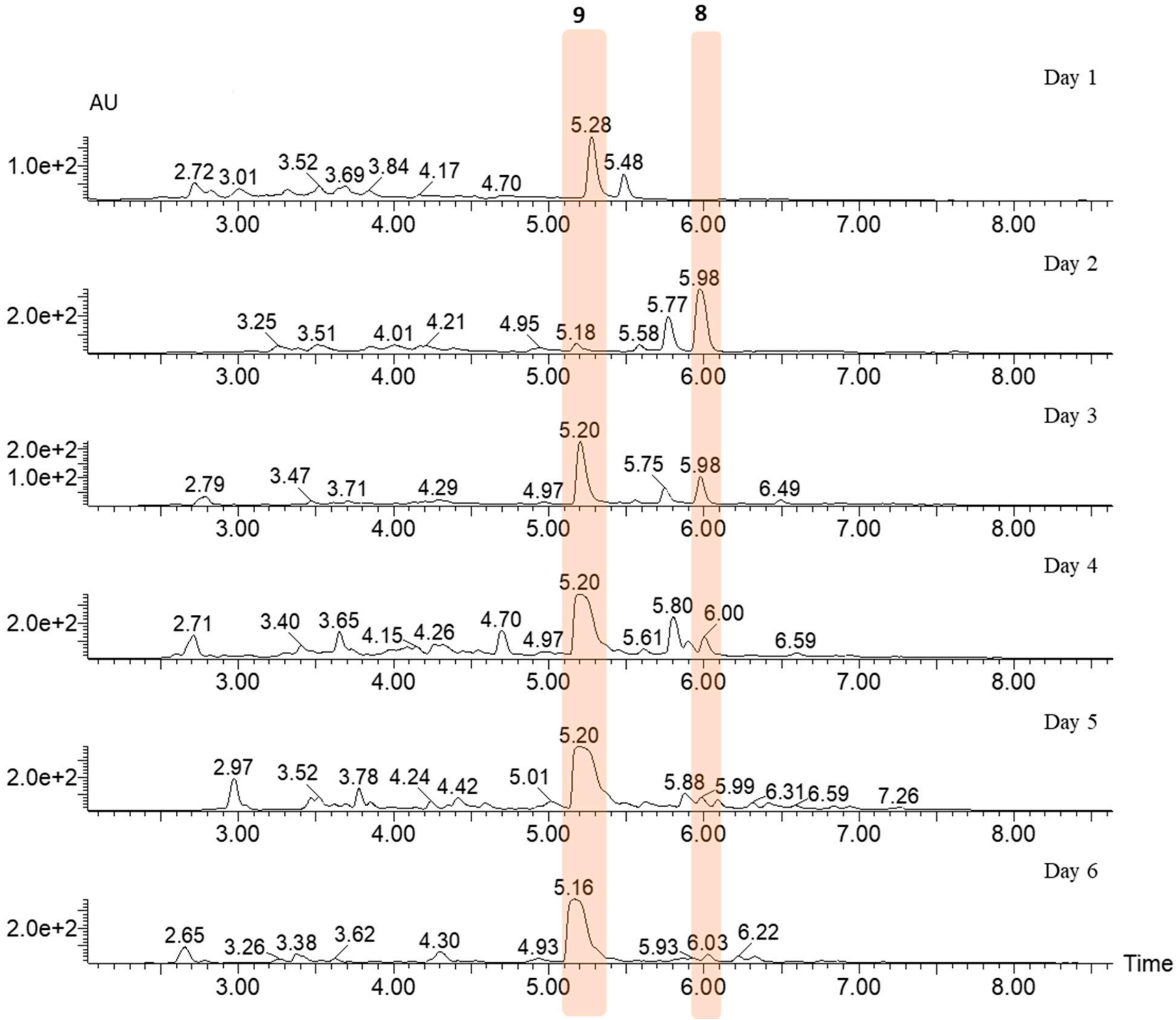

Figure S9.3. Time course for the production of 3-methylorcinaldehyde **8** and 3-methylorsellenic acid **9** from *T. reesei* QM6a·*Δtmus53*·*ΔsorBC*·*P*$_{pdc}$·*aspks1*.

The best producing strain *T. reesei* QM6a·*Δtmus53*·*ΔsorBC*·*P*$_{pdc}$·*aspks1*-C was then cultivated in 1 L DPY + 1 % glucose media (10 x 500 ml flasks) for 48 h at 28 °C and 110 rpm. The extract was used to isolate the two main products of *aspks1* expression, 3-methylorcinaldehyde **8** (4 mg) and 3-methylorsellinic acid **9** (19 mg). The chemical structures of the compounds were confirmed by HRMS and NMR analysis and comparison to published data and the compound with nominal mass 182 was identified as 3-methylorsellenic acid **9**. [16,17]

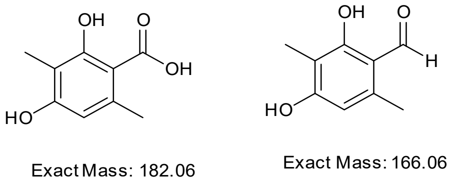

9 **8**

Figure S9.4. Compounds produced in *T. reesei* QM6a·*Δtmus53*·*ΔsorBC*·*P*$_{pdc}$·*aspks1*.

To check the success rate of the transformation, all of the eight transformants obtained were cultivated on PDB media for 48 h. Extraction of all of the transformants showed that seven out of the eight transformants produce 3-methylorcinaldehyde **8**, which represents ~ 87 % success rate. Analysis of the gDNA of all of the eight transformants, showed the correct insertion of the *aspks1* gene in the gDNA of seven out of the eight transformants (Figure S9.5), which is in agreement with the LCMS chromatograms of the transformants.

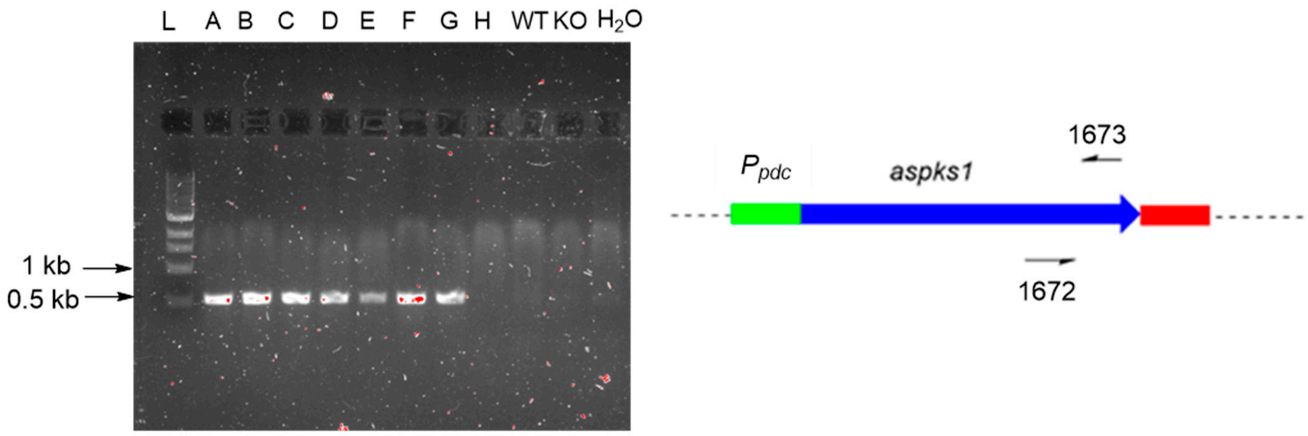

Figure S9.5. PCR analysis for the correct integration of *aspks1* gene in the gDNA of *T. reesei* QM6a·*Δtmus53·ΔsorBC·P_{pdc}·aspks1* colonies A-H using primers 1672 and 1673. The gDNA of *T. reesei* QM6a·*Δtmus53·Δpyr4* (WT) and *T. reesei* QM6a·*Δtmus53·Δpyr4·ΔsorBC* (KO) and H_2O were used as negative controls.

The best producing strain *T. reesei* QM6a·*Δtmus53·ΔsorBC·P_{pdc}·aspks1* was then used for further investigation to test the activity of P_{pdc} in different media. The media used were DPY, DPY + 1 % glucose, PDB, ME (BF) and MA + 1 % glucose (100 ml in 500 ml baffled flasks). On the third day of cultivation (28 °C, 110 rpm), LCMS chromatograms of the transformants showed different levels of production of 3-methylorcinaldehyde **8** and other metabolites of the *aspks1* gene in different media (Figure S9.6). The highest production level of **8** was in DPY + 1 % glucose media, but all the media showed some level of production of *aspks1* metabolites which is expected since P_{pdc} is a constitutive promoter and therefore active under different cultivation conditions.

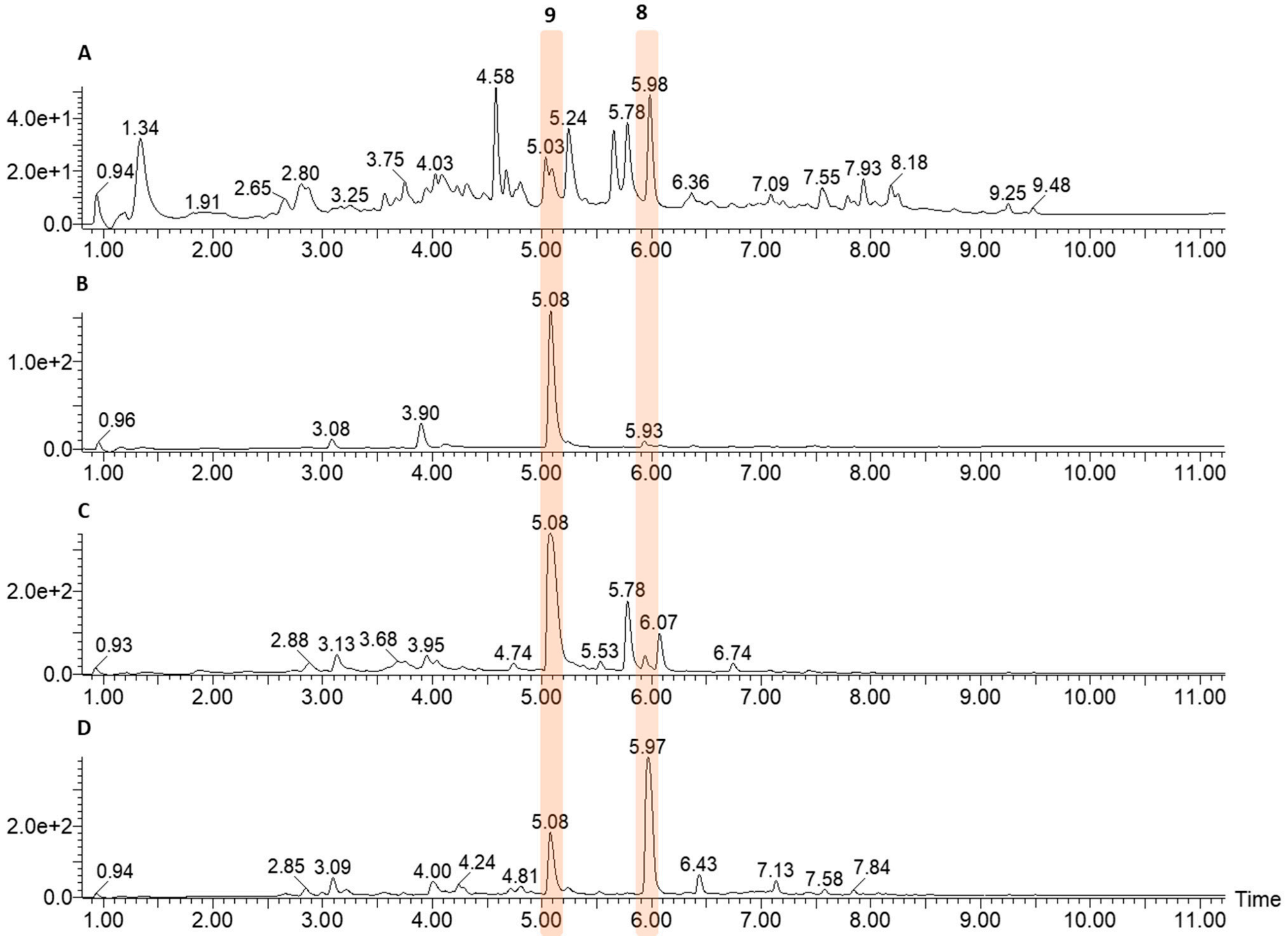

Figure S9.6 DAD chroamtograms of *T. reesei* QM6a·*Δtmus53·ΔsorBC·P$_{pdc}$·aspks1*-C cultivated on different media: **A**, PDB; **B**, MA+ 1 % glucose; **C**, DPY; **D**, DPY + 1 % glucose, showing different levels of production of **8** and **9**.

9.2. Quantification of 3-Methylorcinaldehyde 8 and 3-Methylorsellenic Acid 9

Solutions of 3-methylorcinaldehyde were prepared at the following concentrations: 150, 100, 50, 25, 12.5, 5, 2, 1, 0.5 µg·ml^{-1}. Aliquots of 30 µL were injected *via* a 20 µL sample loop. For each concentration, the peak at 5.9 min which corresponds to 3-methylorcinaldehyde was integrated at a single wavelength (λ_{max} = 295 nm). The area under the curve of each peak (the average of three technical duplicates) was plotted against the concentration. The resulting calibration curve had an R^2 value of 0.9999 and the equation was used to calculate the concentration of 3-methylorcinaldehyde in the media (figure S9.7).

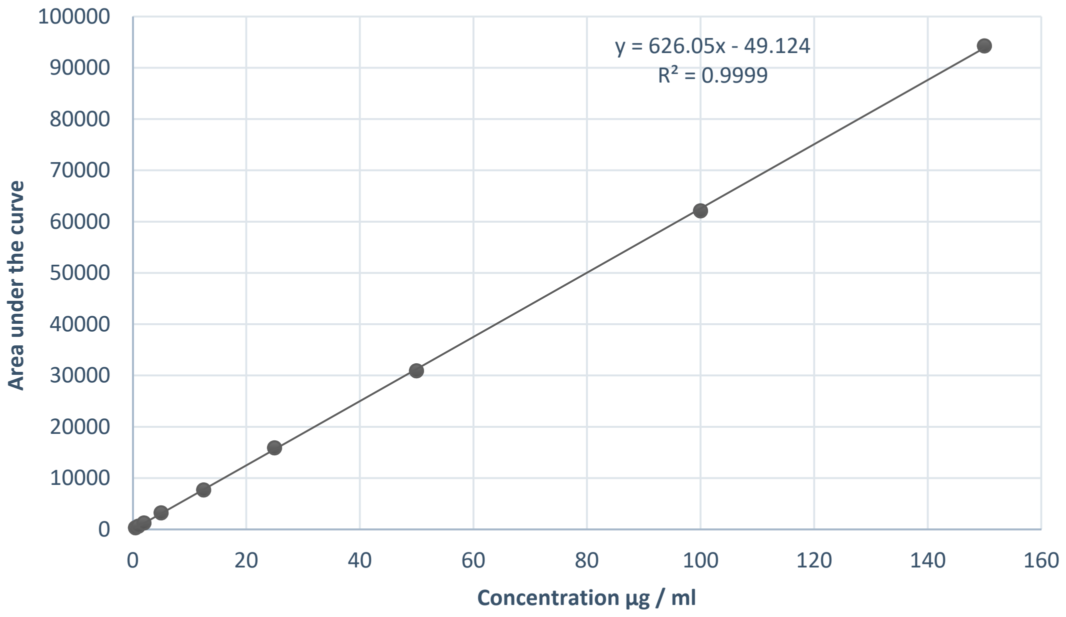

Figure S9.7. Calibration curve of 3-methylorcinaldehyde **8**.

Solutions of 3-methylorsellenic acid **9** were prepared at the following concentrations: 150, 100, 50, 25, 15, 10, 5, 2 µg·ml^{-1}. Aliquots of 30 µL were injected *via* a 20 µL sample loop. For each concentration, the peak at 5.1 min which corresponds to 3-methylorsellenic acid was integrated at a single wavelength (λ_{max} = 265 nm). The area under the curve of each peak (the average of three technical duplicates) was plotted against the concentration. The resulting calibration curve had an R^2 value of 0.9989 and the equation was used to calculate the concentration of 3-methylorsellenic acid in the media (figure S9.8).

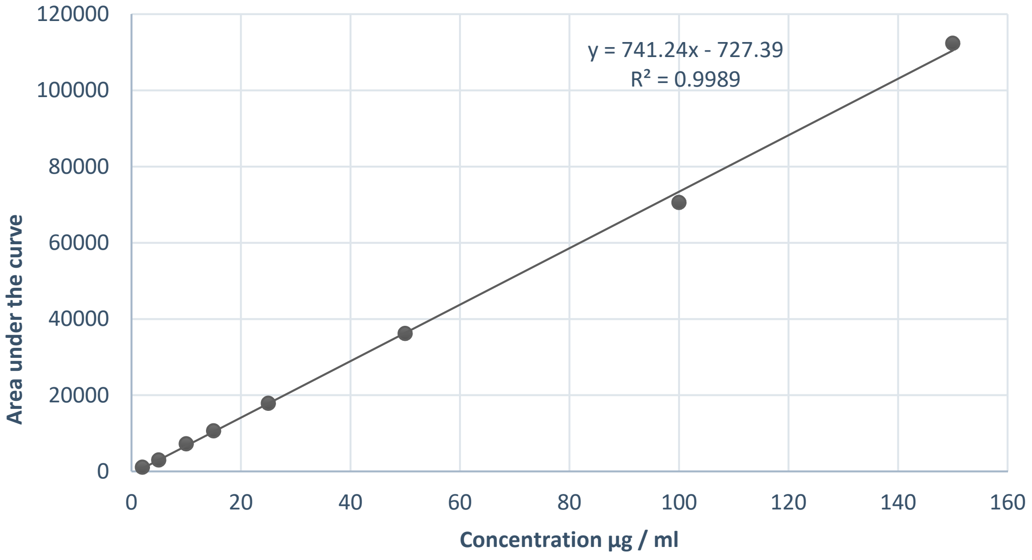

Figure S9.8. Calibration curve of 3-methylorsellenic acid **9**.

9.3. Growth of *T. reesei* QM6a·*Δtmus53*·*ΔsorBC*·P_{pdc}·*aspks1* on Waste Materials

Potato peel, orange peel, banana and kiwi peel (50 g of each) were cut into small pieces and placed in 500 ml flasks with 100 ml water and autoclaved at 121 °C for 15 min. The cold flasks were innoculated with ***T. reesei*** **QM6a·*Δtmus53*·*ΔsorBC*·P_{pdc}·*aspks1***, and *T. reesei* QM6a·*Δtmus53*·*Δpyr4*·*ΔsorBC* as a control, separately. The flasks were incubated without shaking at 28 °C for 14 days in the dark (Figure S9.9). **T**he fermentation mixture was homogenised after 14 days of cultivation using a hand blender and then filtered. The filtrate was then acidified with 2M HCl and extracted with ethyl acetate (2 x 100 ml). LCMS analysis of the extracts showed that ***T. reesei*** **QM6a·*Δtmus53*·*ΔsorBC*·P_{pdc}·*aspks1*** was able to produce **8** and **9** on the three different media but in different ratios (Figure S9.9).

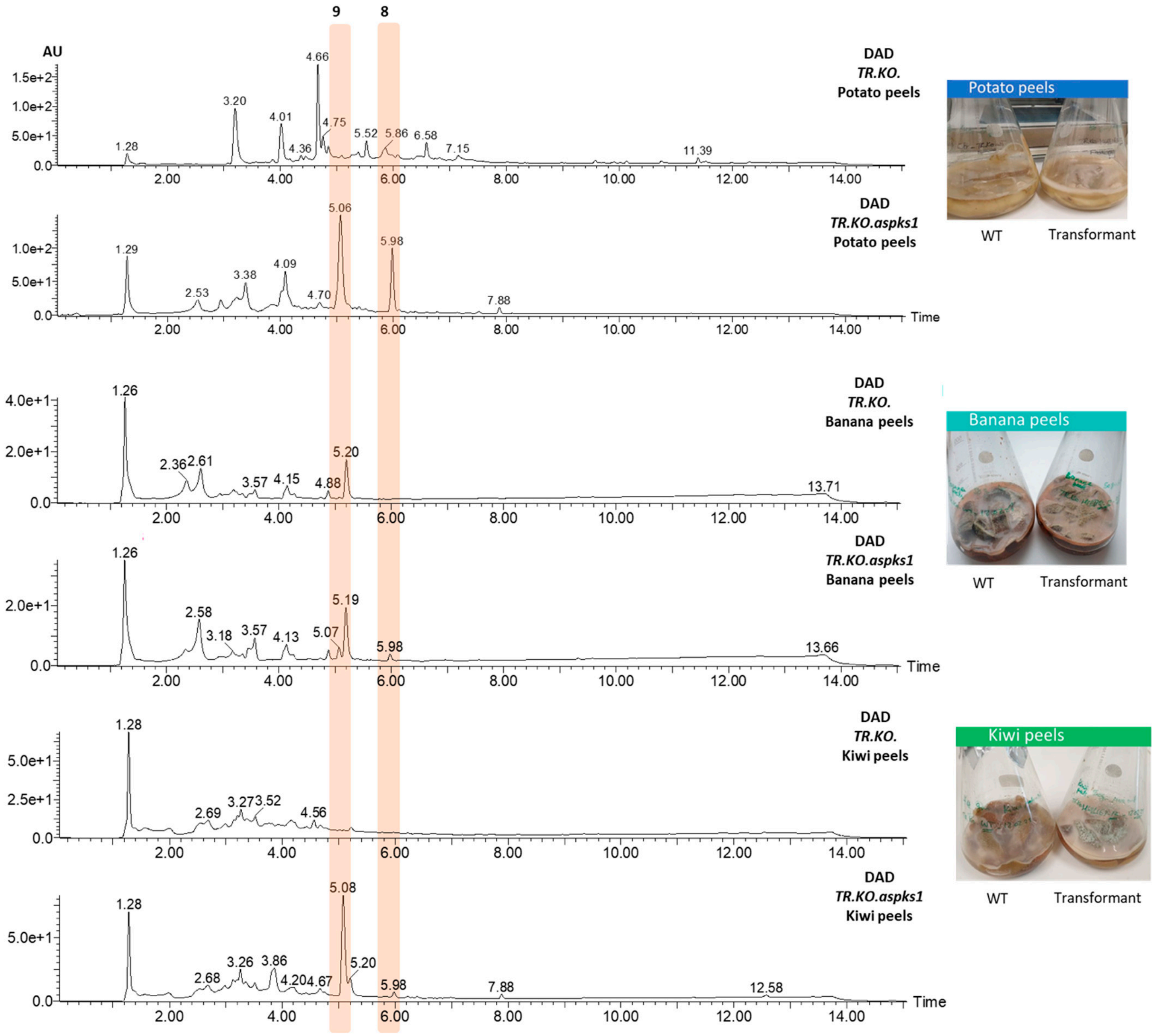

Figure S9.9. DAD LCMS traces of *T. reesei* QM6a·*Δtmus53*·*ΔsorBC*·P_{pdc}·*aspks1-C* transformant growing on different waste materials showing production of 3-methylorcinaldehyde **8** and 3-methylorsellenic acid **9**. *T. reesei* QM6a·*Δtmus53*·*Δpyr4*·*ΔsorBC* strain was used as a control in each case.

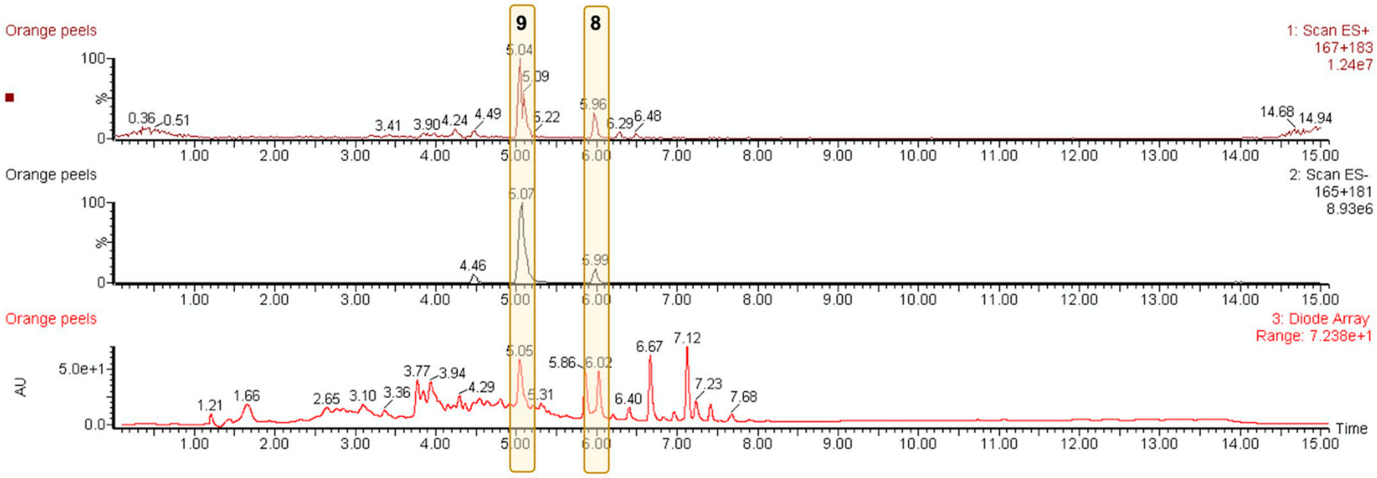

Figure S9.10. DAD LCMS traces of *T. reesei* QM6a·*Δtmus53·ΔsorBC·P_{pdc}·aspks1-C* transformant grown on orange peels showing production of 3-methylorcinaldehyde **8** and 3-methylorsellenic acid **9**. Traces: Top, extracted ion (ES+) chromatogram at *m*/*z* 167 and 183; middle, extracted ion (ES-) chromatogram at *m*/*z* 165 and 181; bottom, Diode array detector 210-600 nm.

10. Expression of *tenS* + *tenC* in *T. reesei* QM6a·*Δtmus53·Δpyr4·ΔsorBC*

Transformation of *T. reesei*·QM6a·*Δtmus53·Δpyr4·ΔsorBC* with pTYGS·*pyr4·P$_{pdc}$·tenS·P$_{cDNA1}$·tenC·P$_{TReno}$* was done using the PEG-mediated transformation of protoplasts and resulted in the production of nine different transformants (A-I). The transformants were selected three times on minimal media and finally transferred into PDA plates. Cultivation of five out of the nine transformants on DPY + 1 % glucose for three days (28 °C / 110rpm) and extraction showed the production of pretenellin A **10** in all of the tested transformants (Figure S10.1).

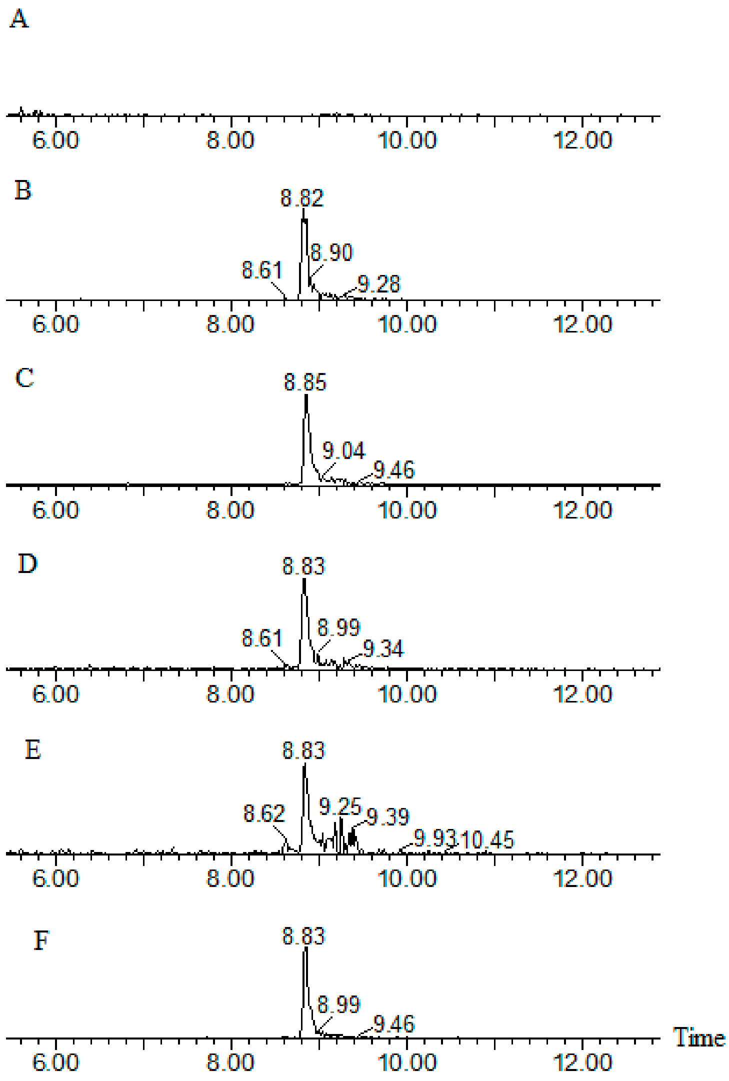

Figure S10.1. EIC (ES-= 354) chromatograms of: **A**, *T. reesei* QM6a·*Δtmus53·ΔsorBC*; **B-F**, different transformants of *T. reesei* QM6a·*Δtmus53·ΔsorBC·P$_{pdc}$·tenS·P$_{cDNA1}$·tenC* on DPY+ 1 % glucose after 3 days of cultivation showing the production of compound **10** at 8.8 min.

PCR analysis of the gDNA of *T. reesei* QM6a·*Δtmus53·ΔsorBC·P$_{pdc}$·tenS·P$_{cDNA1}$·tenC* transformants showed the correct insertion of *tenS* and *tenC* in all of the tested transformants (Figure S10.2). The best producing transformant *T. reesei* QM6a·*Δtmus53·ΔsorBC·P$_{pdc}$·tenS·P$_{cDNA1}$·tenC* was then cultivated on banana peels (50 g autoclaved with 100 ml milipure water) at 28 °C for 14 days without shaking. The LCMS chromatogram of the transfromant after 12 days of growth showed the production of the expected compound **10** (Figure 7-C, D).

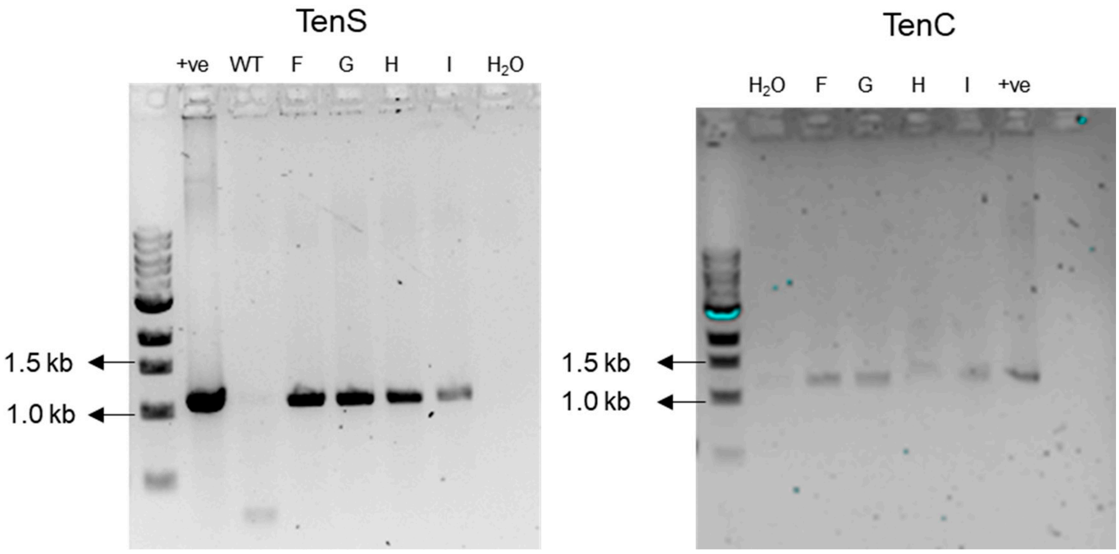

Figure S10.2. Genetic analysis of gDNA of *T. reesei* QM6a·*Δtmus53·ΔsorBC·P_{pdc}·tenS·P_{cDNA1}·tenC* transformants F-I showing the correct integration of *tenS and tenC* genes in all of the tested transformants. Plasmid *pTYGS·pyr4·P_{pdc}·tenS·P_{cDNA1}·tenC·P_{TReno}* was used as positive control and H_2O and/or *T. reesei* QM6a·*Δtmus53·Δpyr4* as a negative control.

10.1 Characterisation of Pretenellin A 10 produced in *T. reesei*.

The biosynthetic genes *tenS* and *tenC* were previously expressed in *A. oryzae* to produce pretenellin A **10**.[18] Extracts from the *A. oryzae* and *T. reesei* expression experiments were compared by LCMS analysis to prove the identity of **10**.

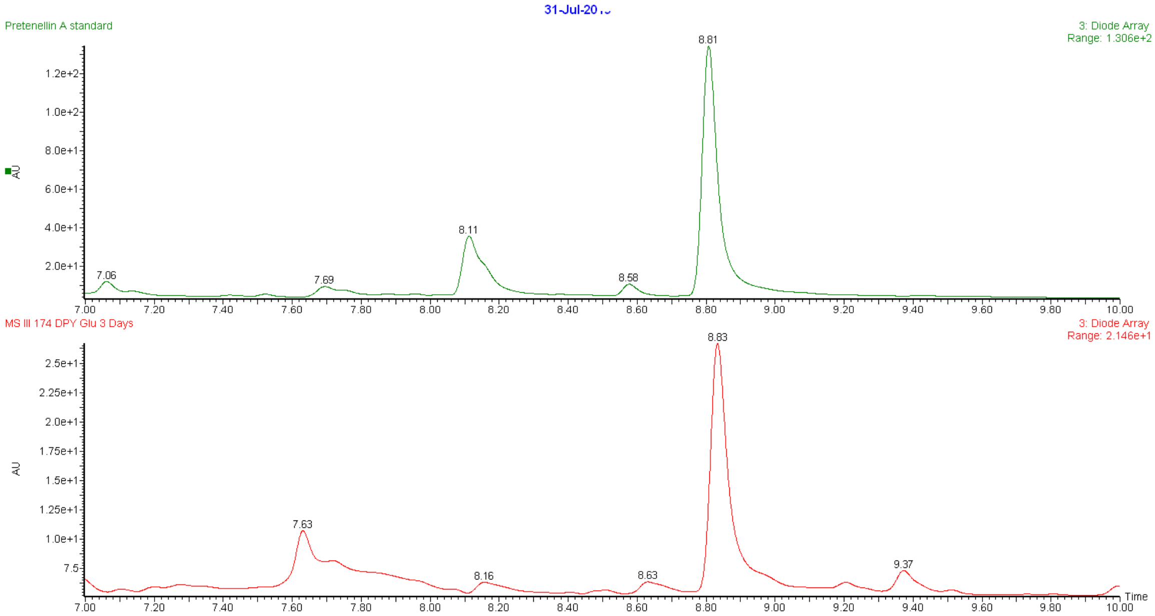

Figure S10.3. Comparison of extract from *A. oryzae tenS·tenC* with extract from *T. reesei* QM6a·*Δtmus53·ΔsorBC·P_{pdc}·tenS·P_{cDNA1}·tenC*. Diode array traces 210-600 nm. Top, *A. oryzae*; Bottom, *T. reesei*.

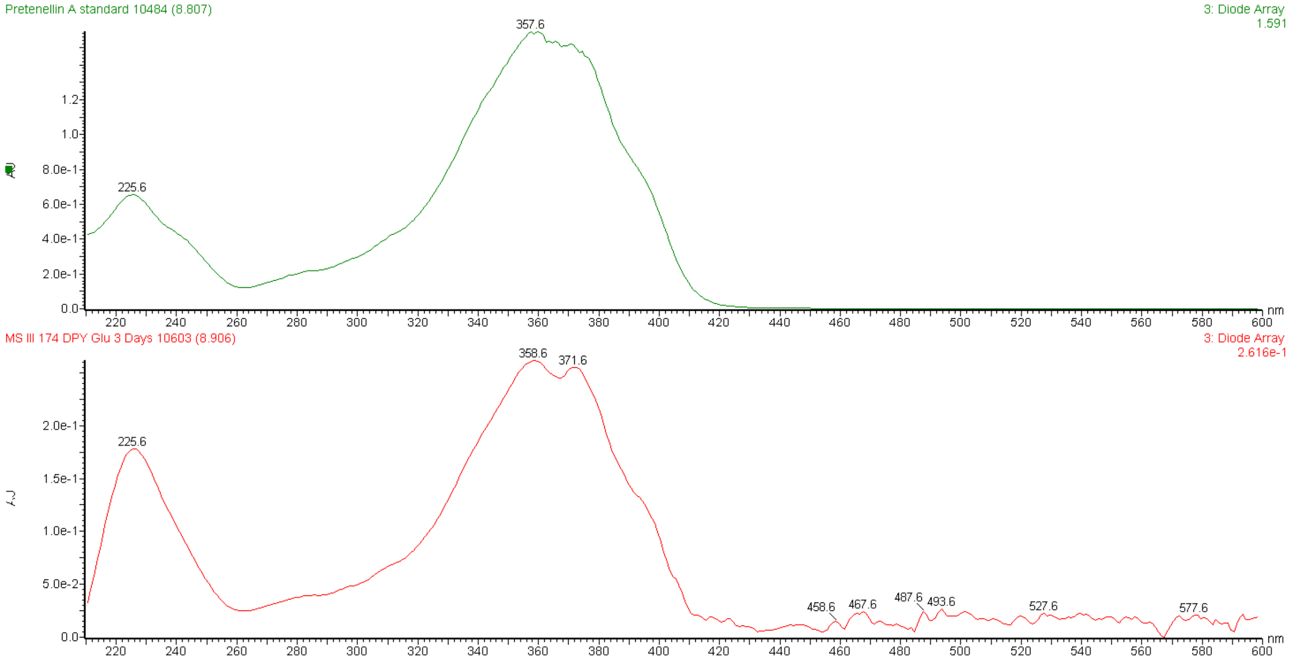

Figure S10.4. Comparison of uv spectra at 8.8 min. Top, *A. oryzae*; Bottom, *T. reesei*.

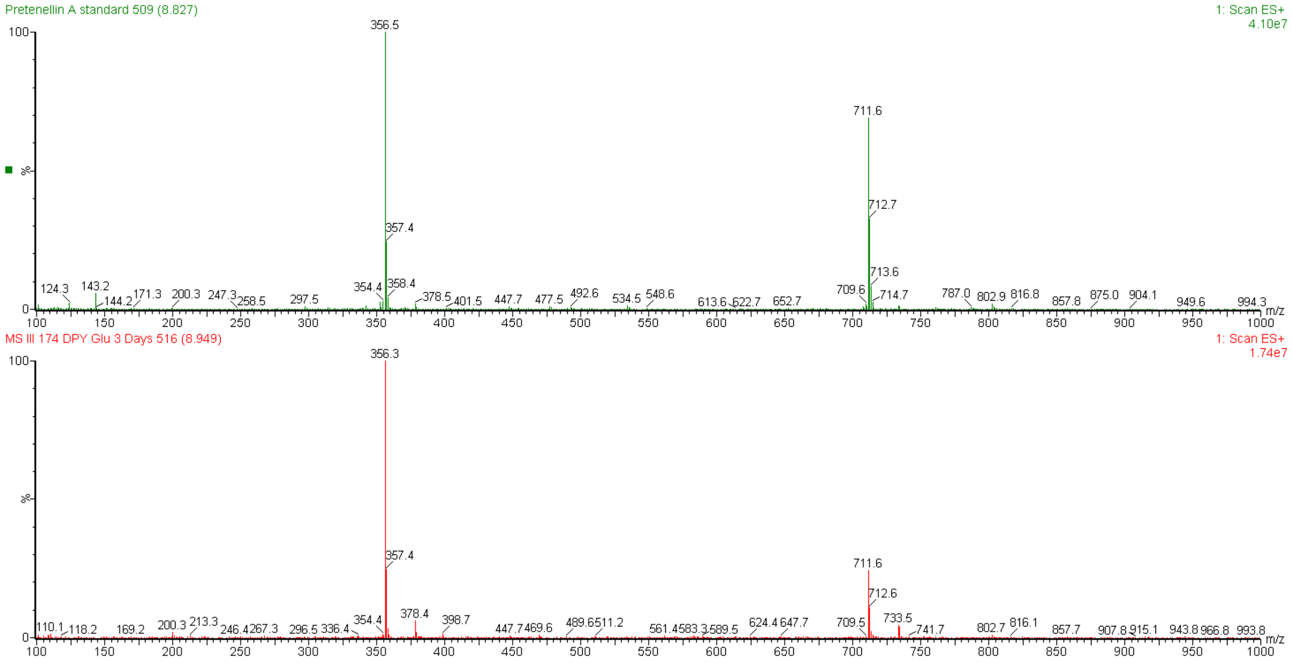

Figure S10.5. Comparison of ES+ spectra at 8.8 min. Top, *A. oryzae*; Bottom, *T. reesei*.

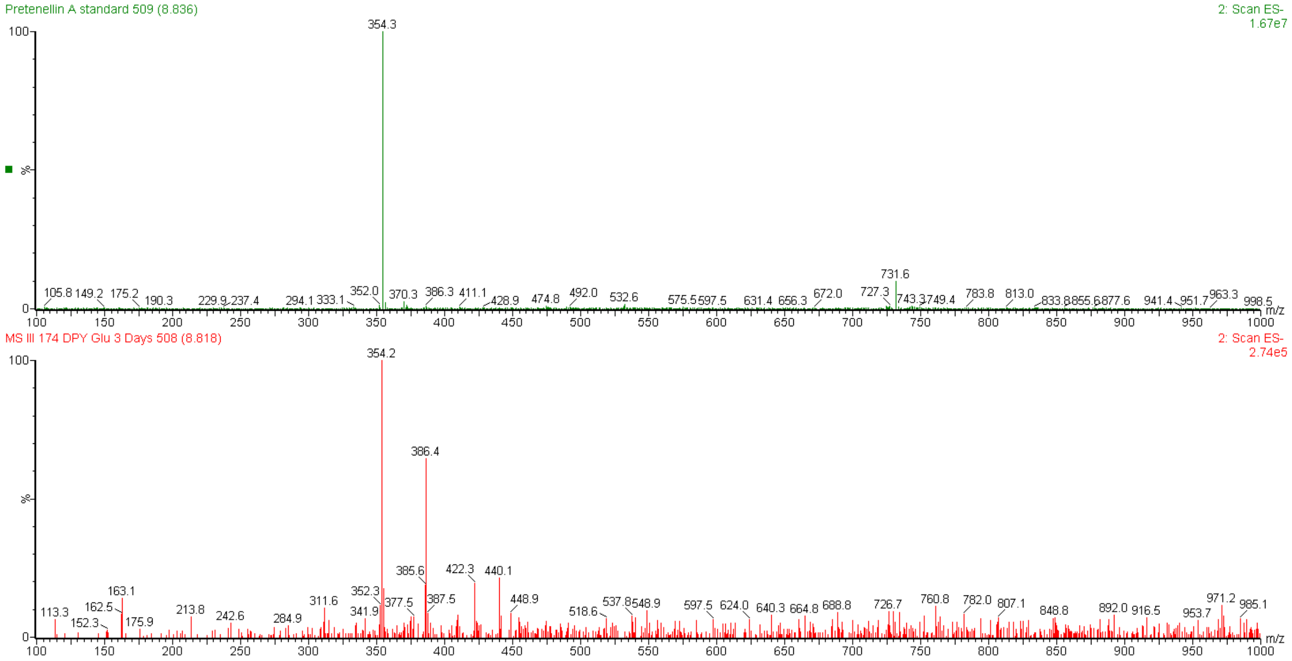

Figure S10.6. Comparison of ES- spectra at 8.8 min. Top, *A. oryzae*; Bottom, *T. reesei*.

10.2 Characterisation of other peaks produced on banana peel

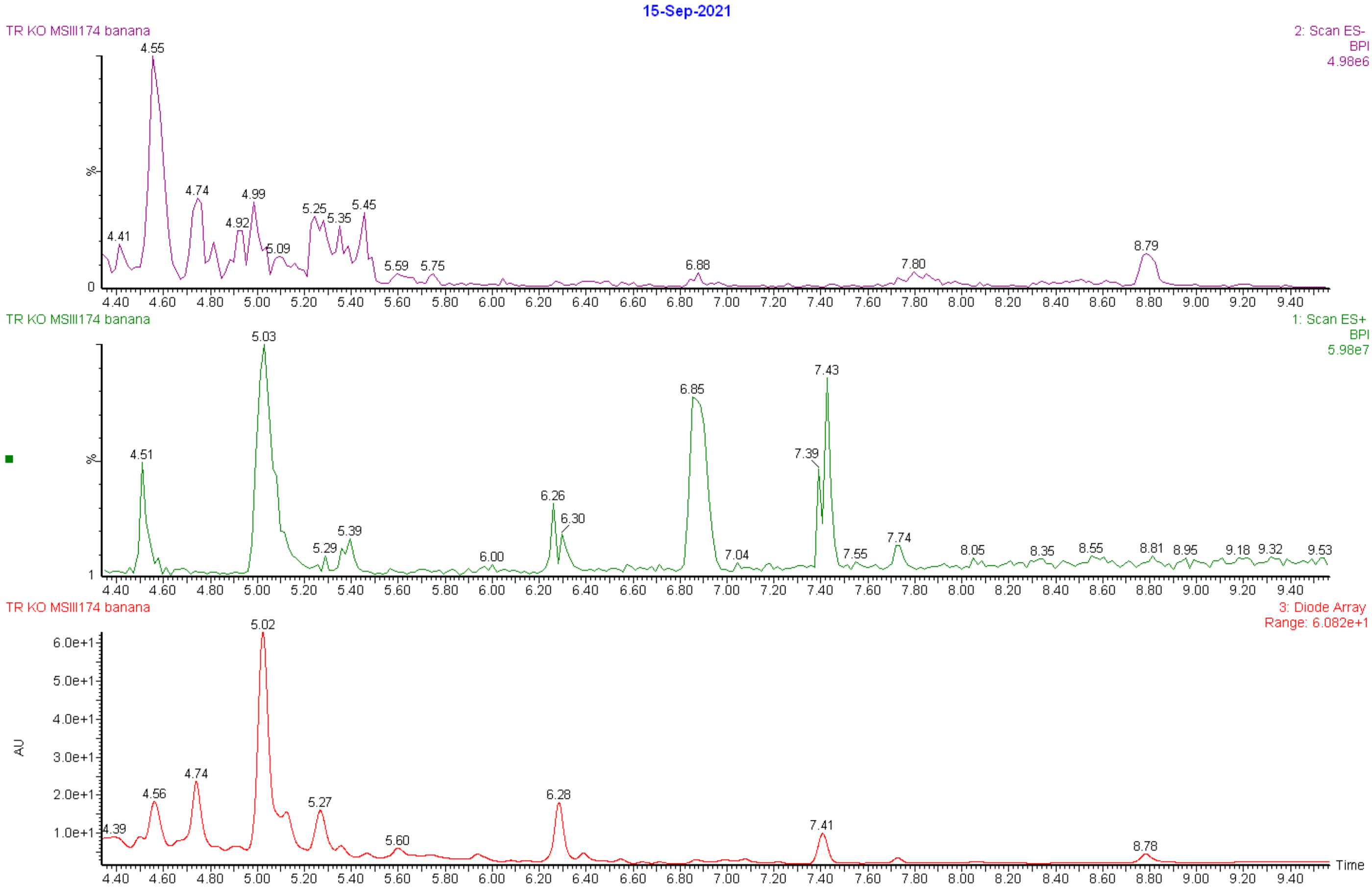

Figure S10.7. Expansion of LCMS data for *T. reesei* QM6a·*Δtmus53*·*ΔsorBC*·*P$_{pdc}$*·*tenS*·*P$_{cDNA1}$*·*tenC* grown on banana peel.

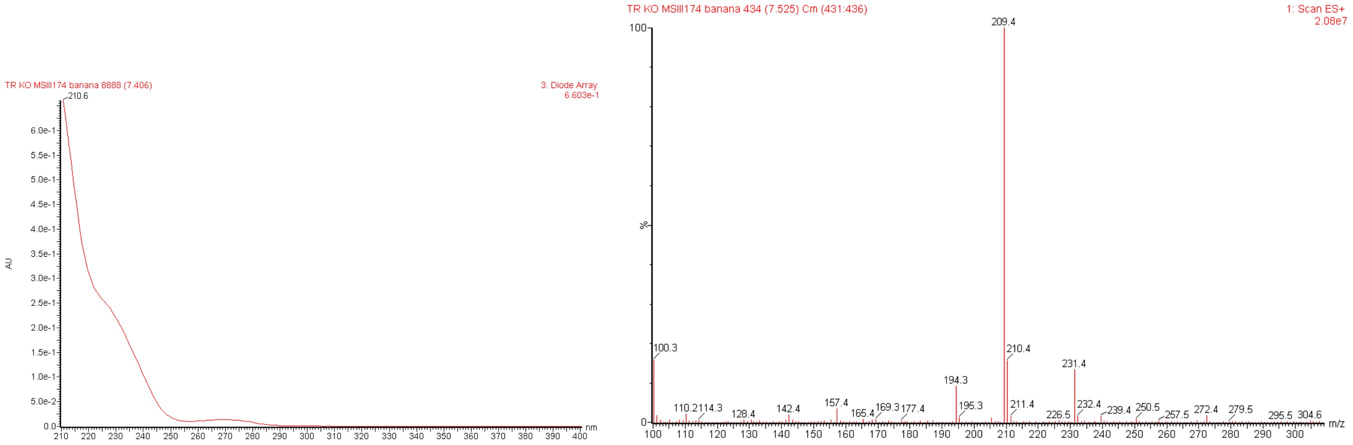

Figure S10.8. Characterisation of 7.4 min peak.

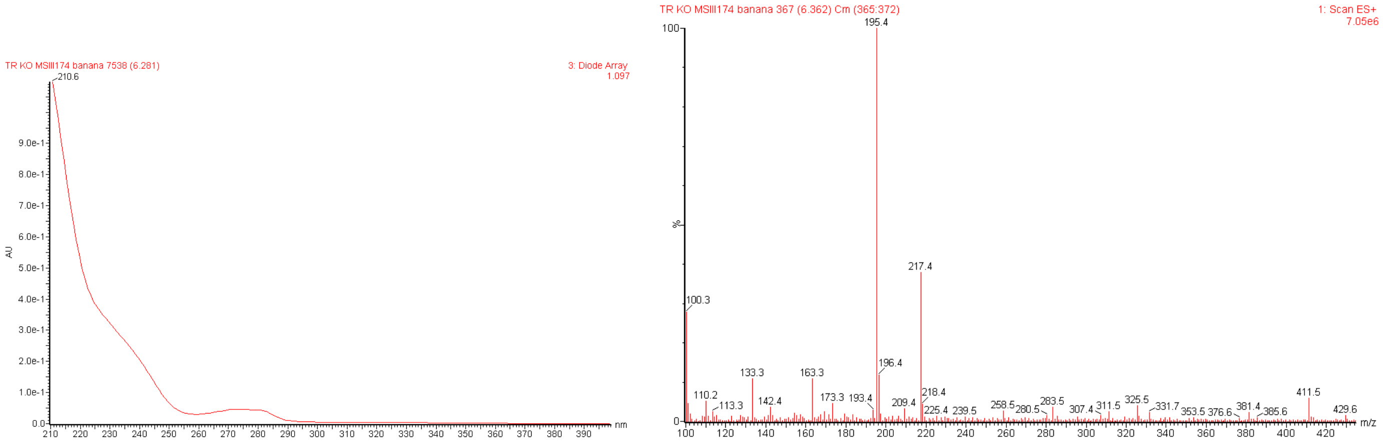

Figure S10.9. Characterisation of 6.3 min peak.

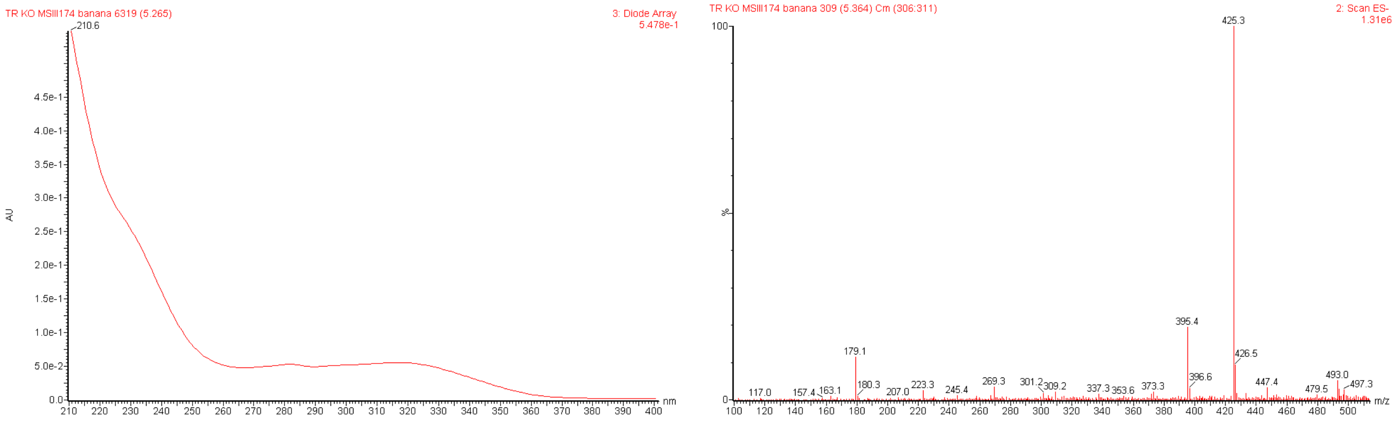

Figure S10.10. Characterisation of 5.3 min peak.

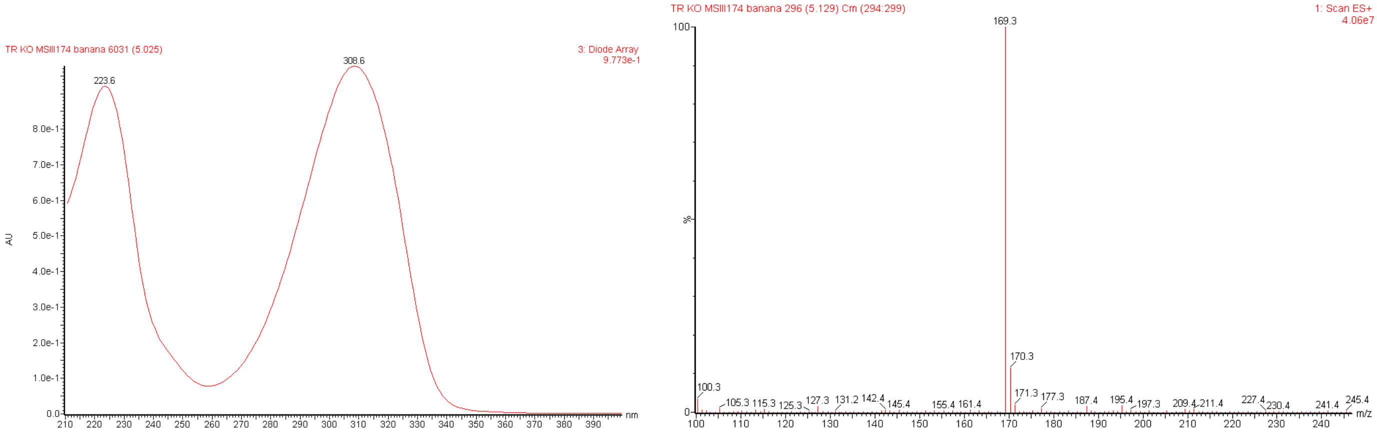

Figure S10.11. Characterisation of 5.0 min peak.

11. Selected NMR data.

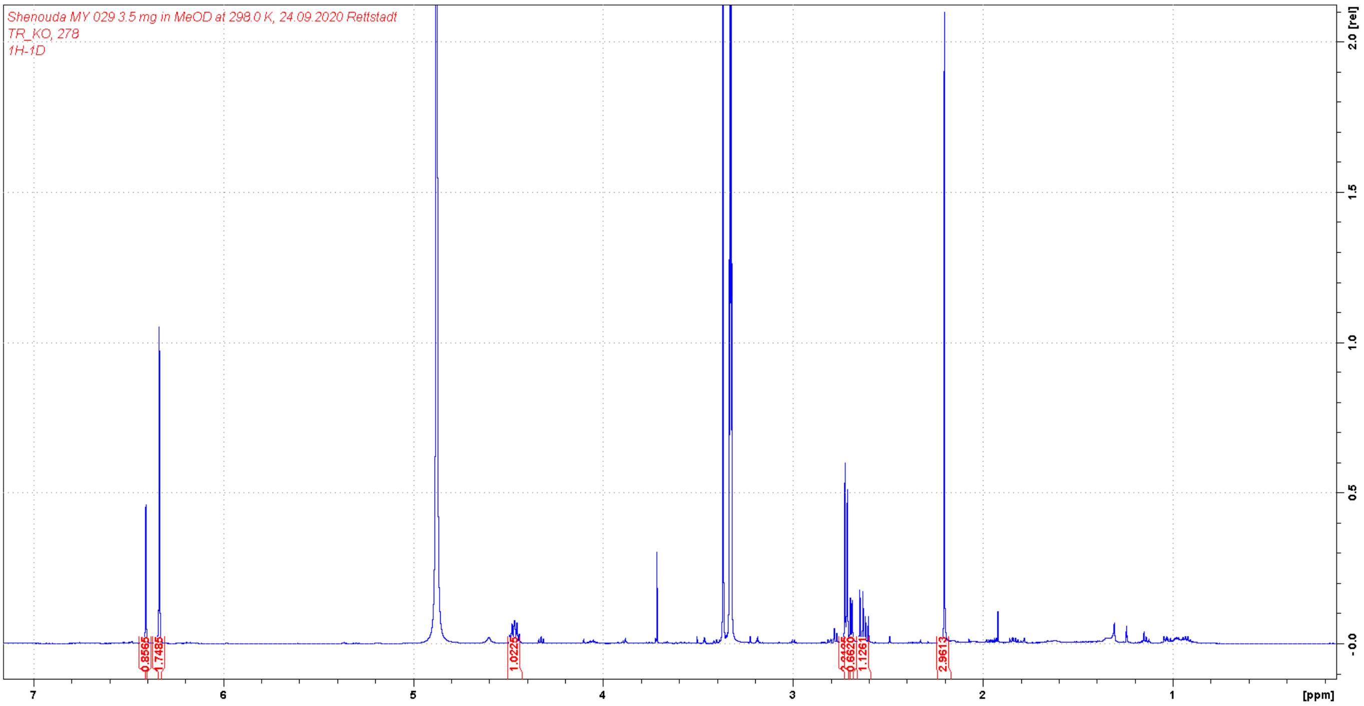

Figure S11.1. ^{1}H-NMR spectrum of compound **7** (citreoisocoumarin, 500 MHz, CD_3OD).

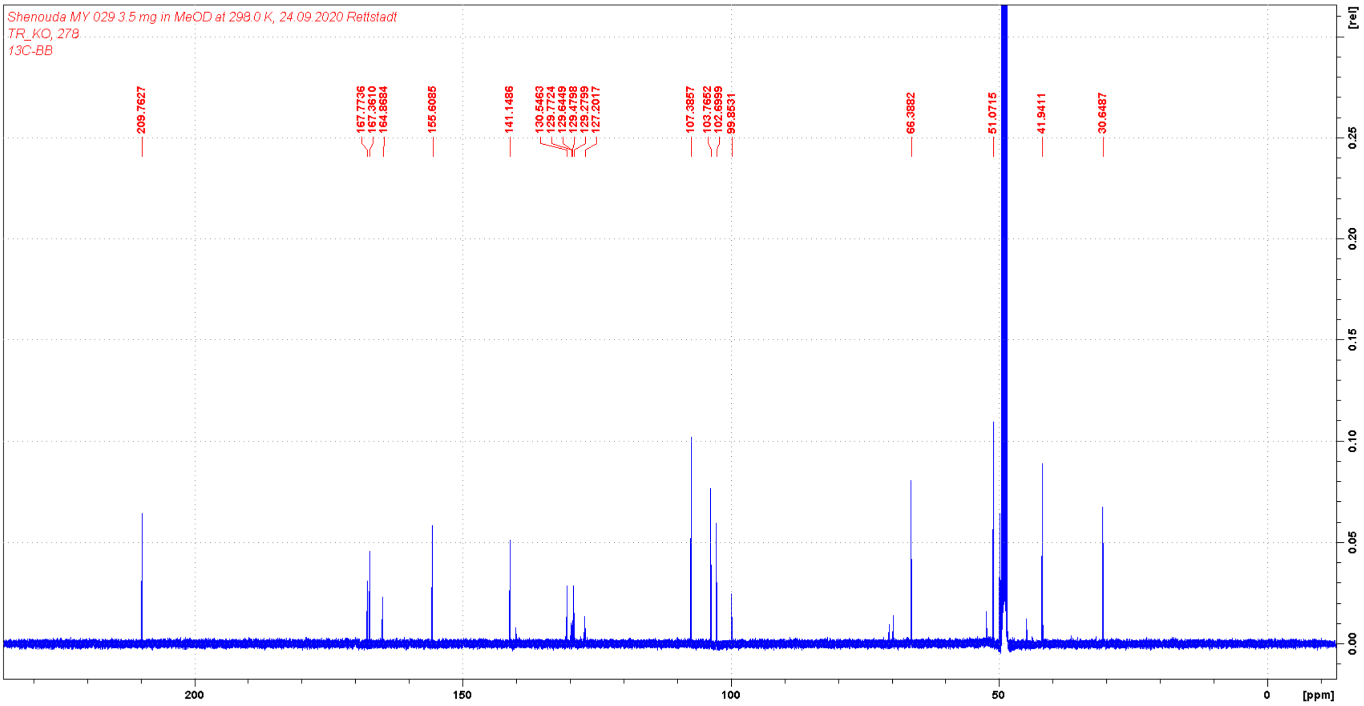

Figure S11.2. ^{13}C-NMR spectrum of compound **7** (Citreoisocoumarin, 125 MHz, CD_3OD).

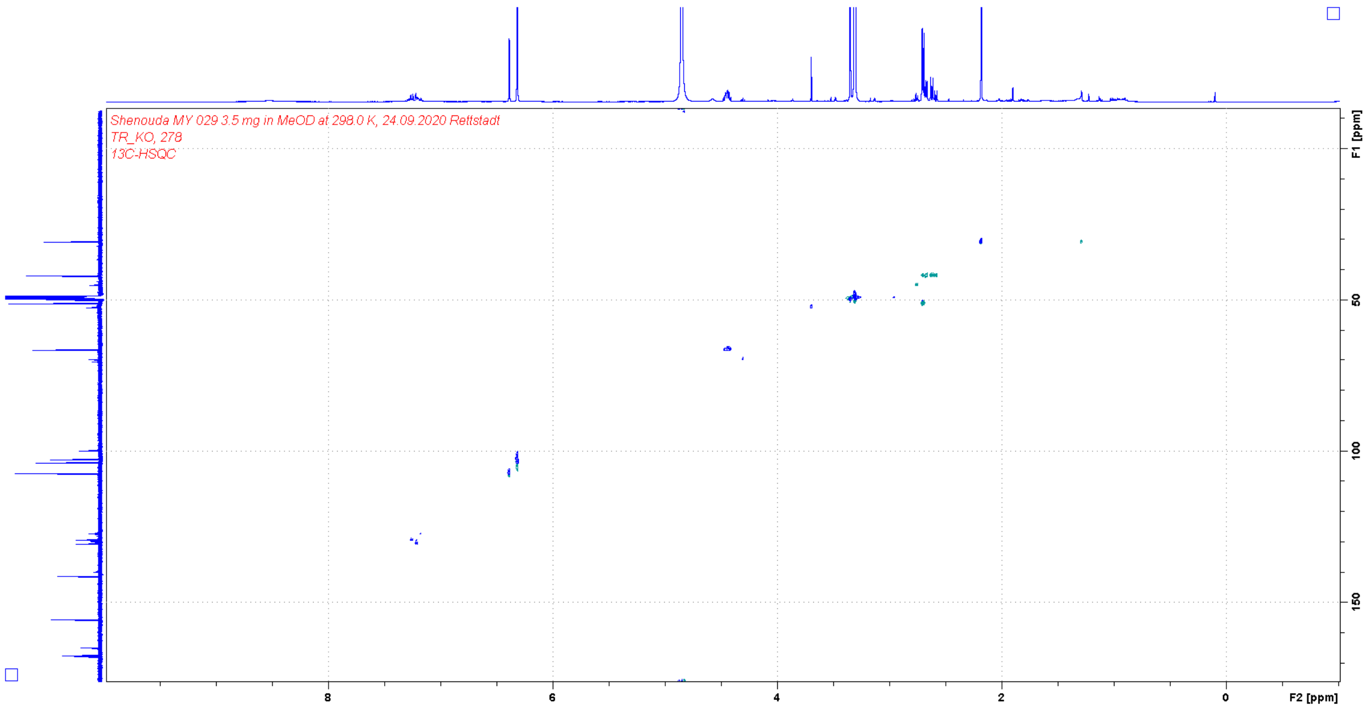

Figure S11.3. HSQC spectrum of compound **7** (Citroisocoumarin).

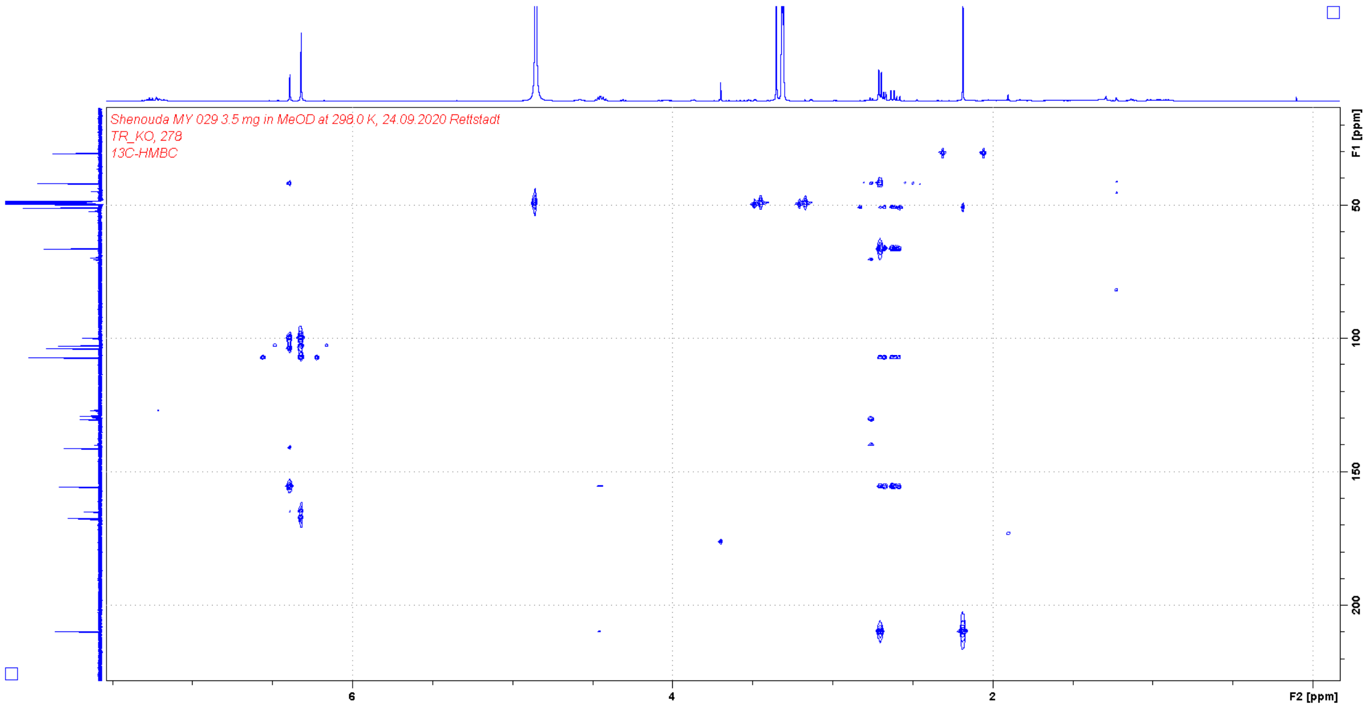

Figure S11.4. HMBC spectrum of compound **7** (Citroisocoumarin).

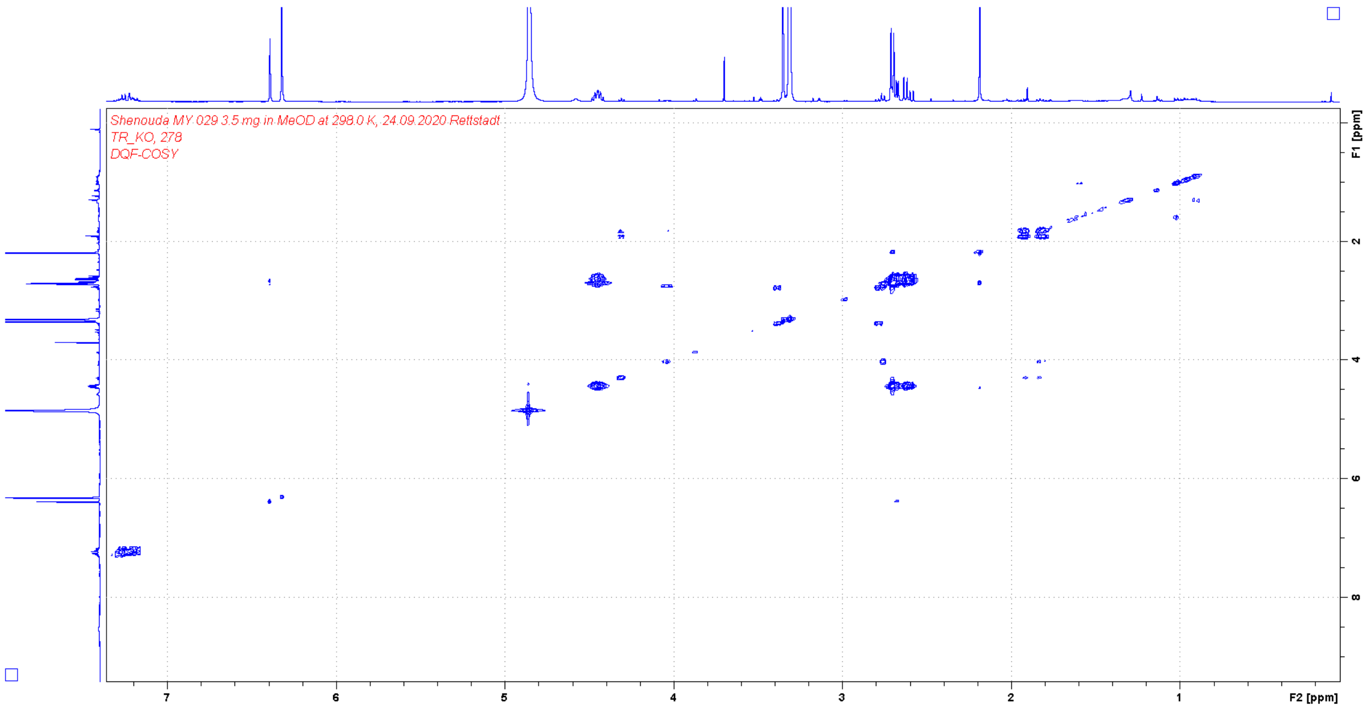

Figure S11.5. COSY spectrum of compound **7** (Citroisocoumarin).

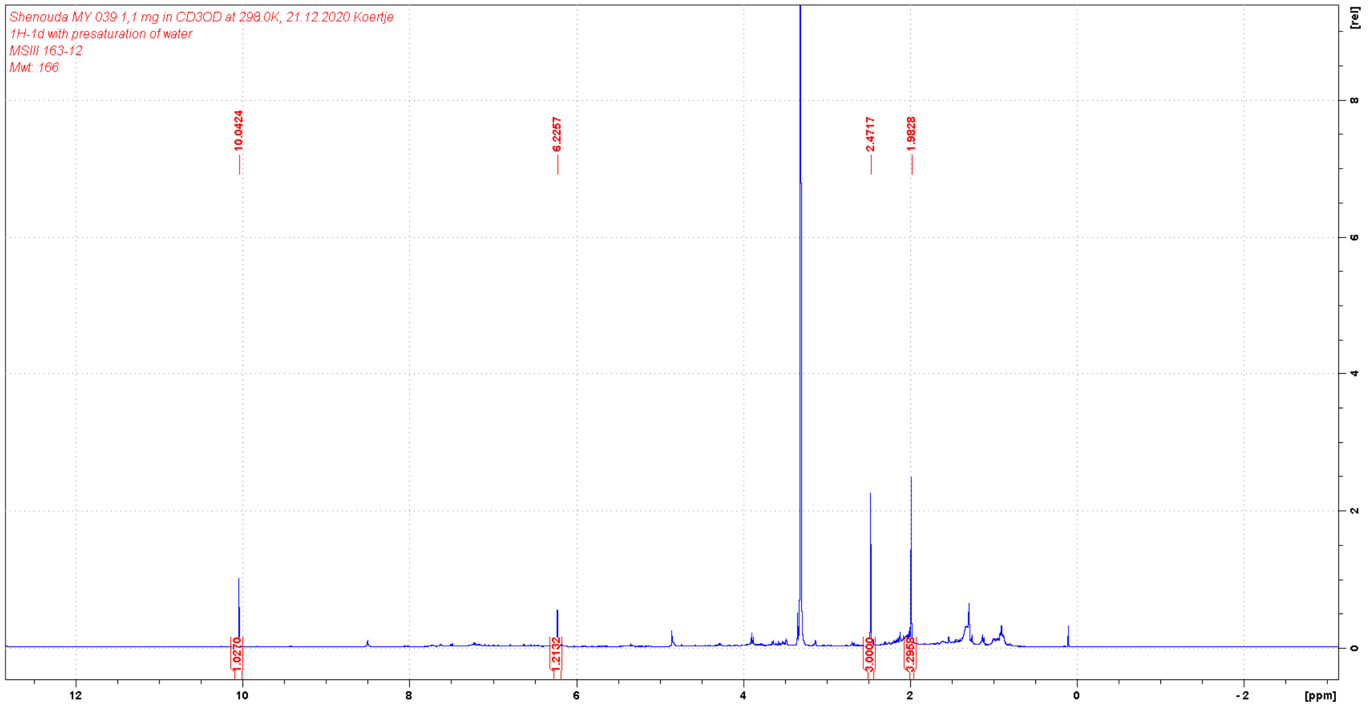

Figure S11.6. ^{1}H-NMR spectrum of compound **8** (3-methylorcinaldehyde, 500 MHz, CD_3OD).

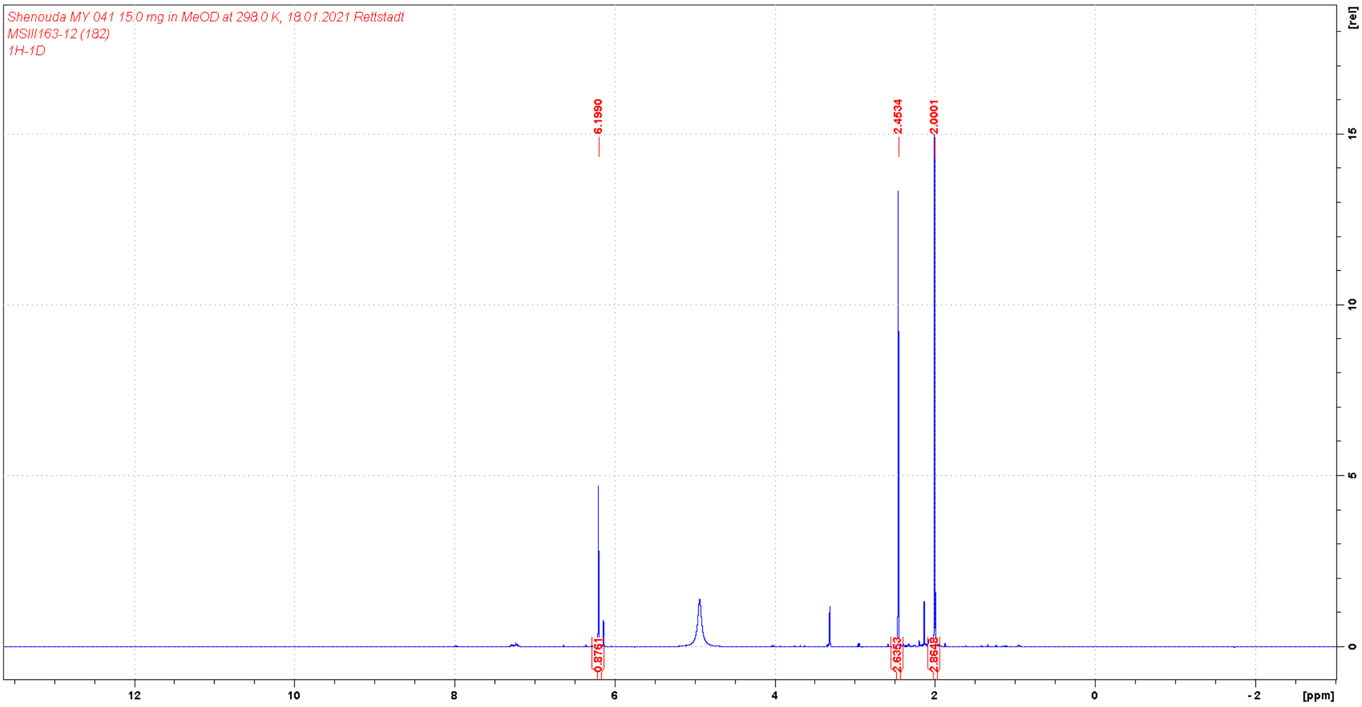

Figure S11.7. ^{1}H-NMR spectrum of compound **9** (3-methylorsellenic acid, 500 MHz, CD_3OD).

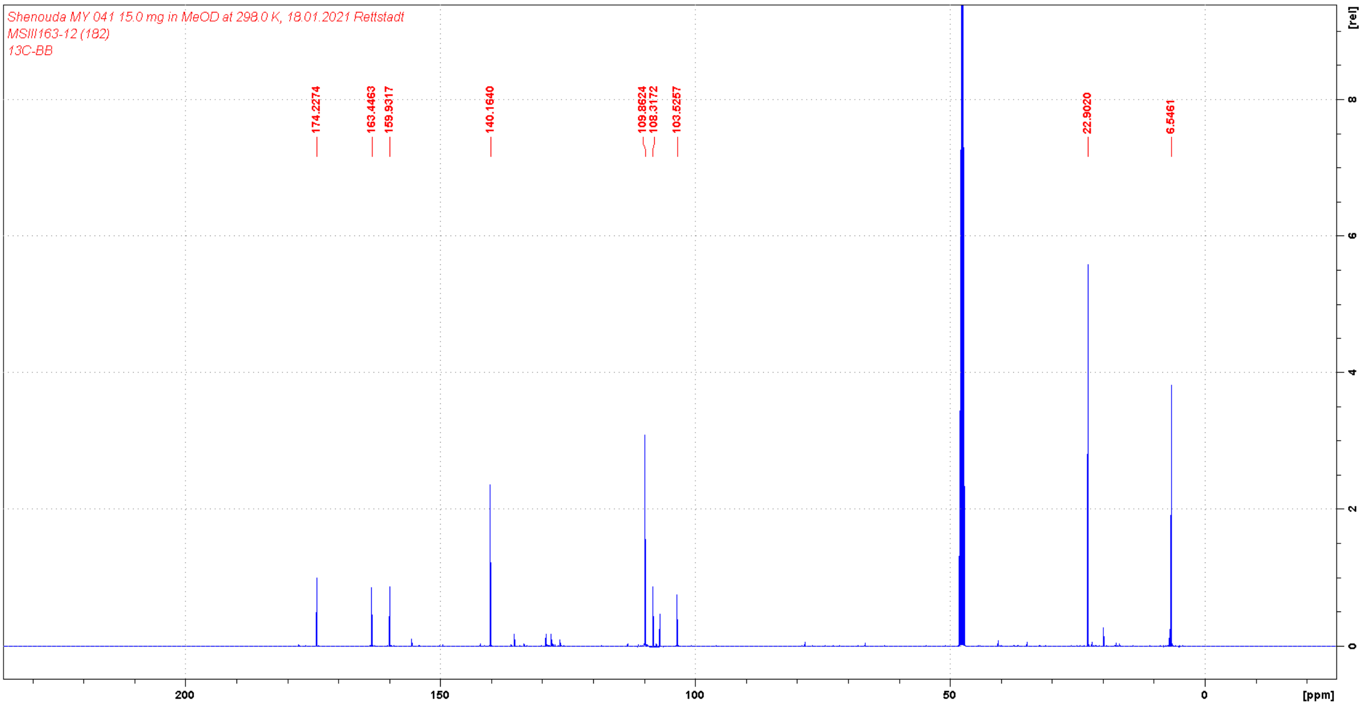

Figure S11.8. ^{13}C-NMR of compound **9** (3-methylorsellenic acid, 125 MHz, CD_3OD).

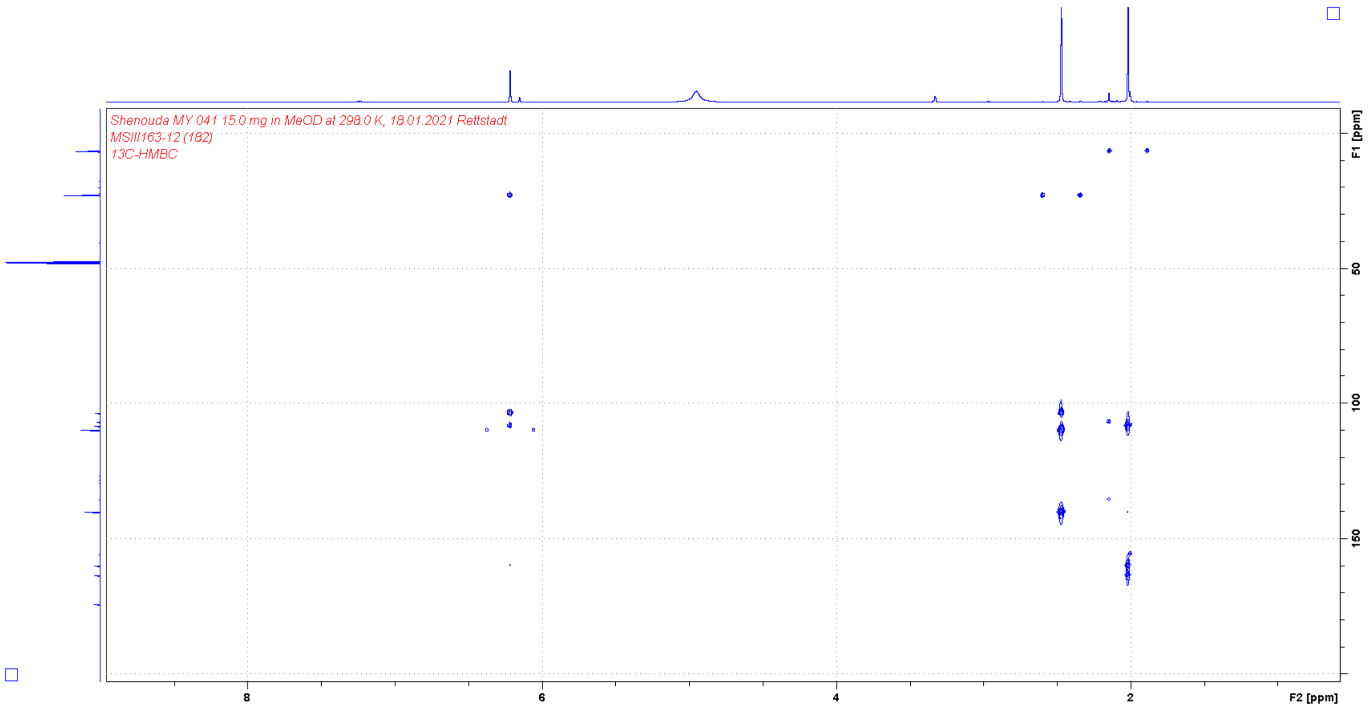

Figure S11.9. HMBC spectrum of compound **9** (3-methylorsellenic acid).

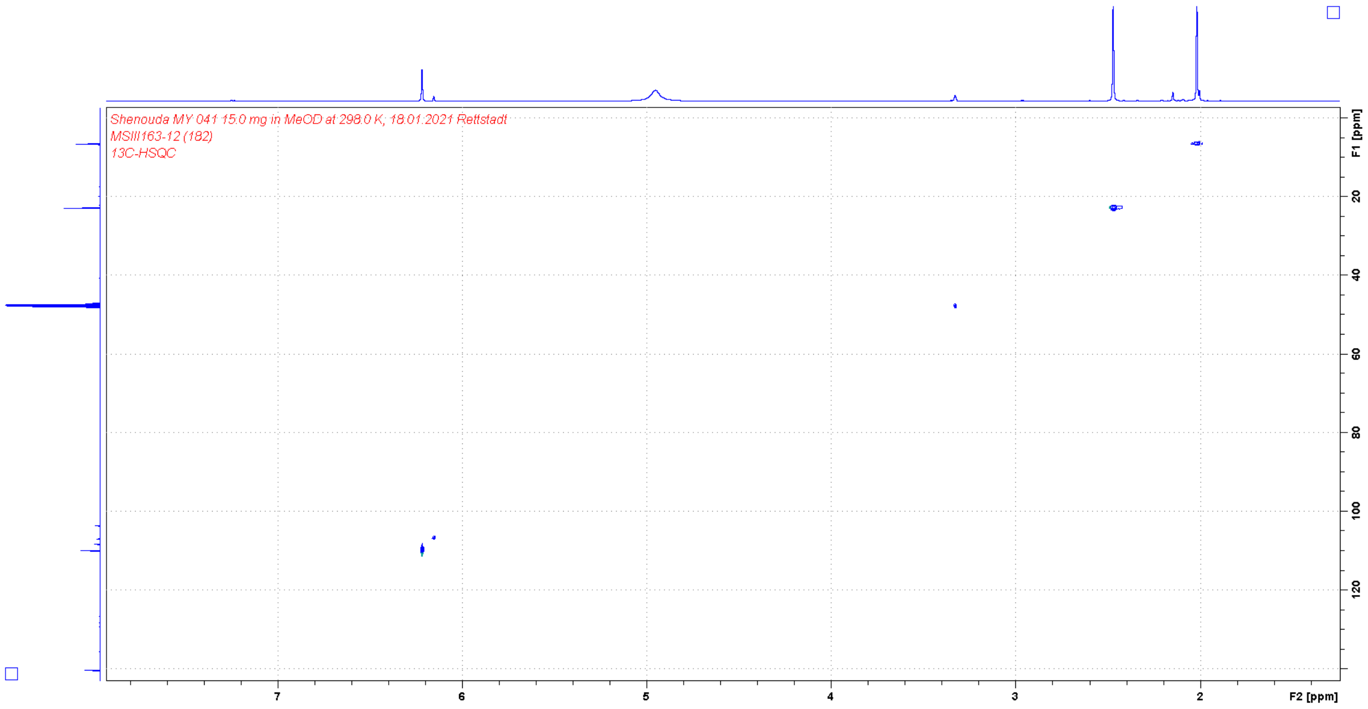

Figure S11.10. HSQC spectrum of compound **9** (3-methylorsellenic acid).

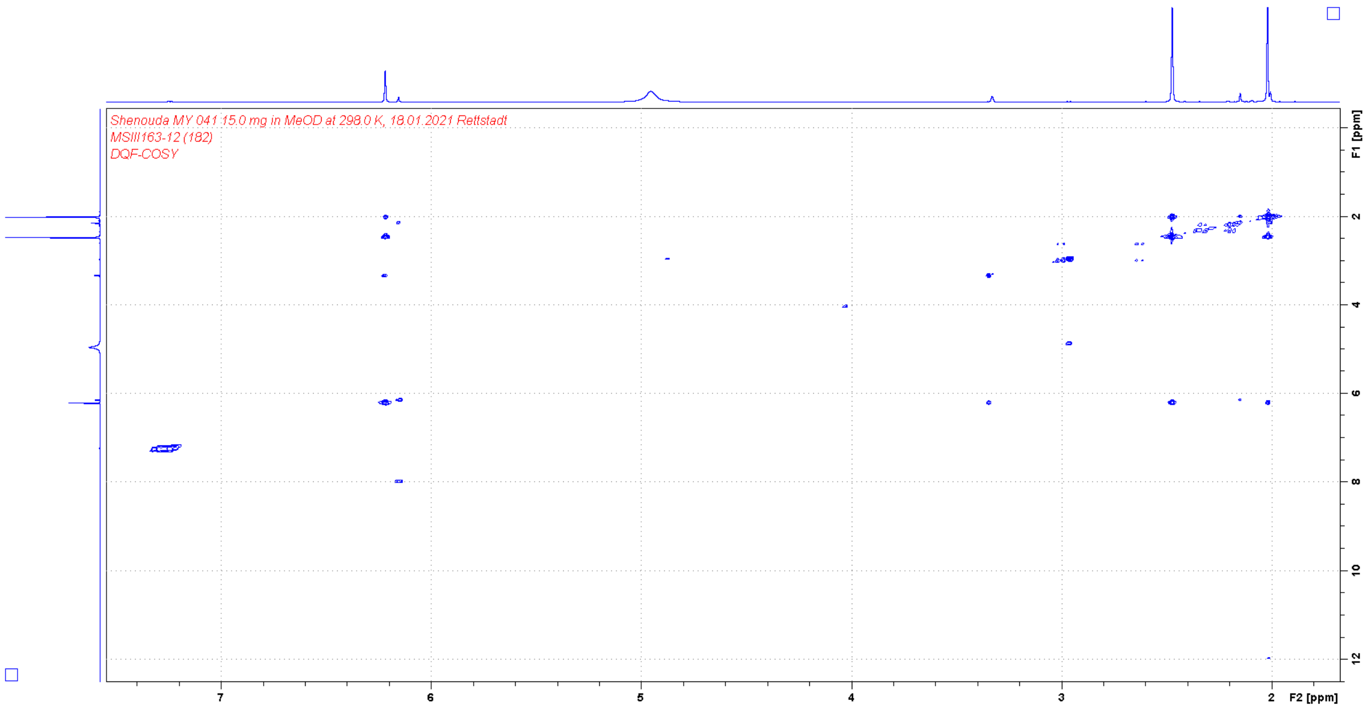

Figure S11.11. COSY spectrum of compound **9** (3-methylorsellenic acid).

12. References

1. Van Dijken, J.P.; Bauer, J.; Brambilla, L.; Duboc, P.; Francois, J.M.; Gancedo, C.; Giuseppin, M.L.F.; Heijnen, J.J.; Hoare, M.; Lange, H.C.; et al. An interlaboratory comparison of physiological and genetic properties of four Saccharomyces cerevisiae strains. *Enzyme Microb. Technol.* **2000**, *26*, 706–714, doi:10.1016/S0141-0229(00)00162-9.
2. Derntl, C.; Kiesenhofer, D.P.; Mach, R.L.; Mach-Aigner, A.R. Novel Strategies for Genomic Manipulation of Trichoderma reesei with the Purpose of Strain Engineering. *Appl. Environ. Microbiol.* **2015**, *81*, 6314–6323, doi:10.1128/AEM.01545-15.
3. Pahirulzaman, K. a. K.; Williams, K.; Lazarus, C.M. *A toolkit for heterologous expression of metabolic pathways in aspergillus oryzae*; 1st ed.; Elsevier Inc., 2012; Vol. 517; ISBN 9780124046344.
4. Schor, R.; Schotte, C.; Wibberg, D.; Kalinowski, J.; Cox, R.J. Three previously unrecognised classes of biosynthetic enzymes revealed during the production of xenovulene A. *Nat. Commun.* **2018**, *9*, 1–9, doi:10.1038/s41467-018-04364-9.
5. Xiao-Long Yang; Steffen Friedrich; Sen Yin; Oliver Piech; Katherine Williams; J. Simpson, T.; J. Cox, R. Molecular basis of methylation and chain-length programming in a fungal iterative highly reducing polyketide synthase. *Chem. Sci.* **2019**, *10*, 8478–8489, doi:10.1039/C9SC03173A.
6. Gietz, R.D.; Schiestl, R.H. High-efficiency yeast transformation using the LiAc/SS carrier DNA/PEG method. *Nat. Protoc.* **2007**, *2*, 31–34, doi:10.1038/nprot.2007.13.
7. Gruber, F.; Visser, J.; Kubicek, C.P.; de Graaff, L.H. The development of a heterologous transformation system for the cellulolytic fungus Trichoderma reesei based on a pyrG-negative mutant strain. *Curr. Genet.* **1990**, *18*, 71–76, doi:10.1007/BF00321118.
8. Dockrey, S.A.B.; Narayan, A.R.H. Photocatalytic Oxidative Dearomatization of Orcinaldehyde Derivatives. *Org. Lett.* **2020**, *22*, 3712–3716, doi:10.1021/ACS.ORGLETT.0C01207.
9. Shang, Z.; Khalil, Z.; Li, L.; Salim, A.A.; Quezada, M.; Kalansuriya, P.; Capon, R.J. Roseopurpurins: Chemical Diversity Enhanced by Convergent Biosynthesis and Forward and Reverse Michael Additions. *Org. Lett.* **2016**, *18*, 4340–4343, doi:10.1021/ACS.ORGLETT.6B02099.
10. Hantke, V.; Wang, C.; Skellam, E.J.; Cox, R.J. Function of pathway specific regulators in the ACE1 and pyrichalasin H biosynthetic gene clusters. *RSC Adv.* **2019**, *9*, 35797–35802, doi:10.1039/C9RA07028A.
11. Li, J.; Wang, J.; Wang, S.; Xing, M.; Yu, S.; Liu, G. Achieving efficient protein expression in Trichoderma reesei by using strong constitutive promoters. *Microb. Cell Fact.* **2012**, *11*, 84, doi:10.1186/1475-2859-11-84.
12. Uzbas, F.; Sezerman, U.; Hartl, L.; Kubicek, C.P.; Seiboth, B. A homologous production system for Trichoderma reesei secreted proteins in a cellulase-free background. *Appl. Microbiol. Biotechnol.* **2012**, *93*, 1601–1608, doi:10.1007/S00253-011-3674-8.
13. Nakari-Setala, T.; Penttila, M. Production of Trichoderma reesei cellulases on glucose-containing media. *Appl. Environ. Microbiol.* **1995**, *61*, 3650–3655, doi:10.1128/AEM.61.10.3650-3655.1995.
14. Fang, F.; Zhao, J.; Ding, L.; Huang, C.; Naman, C.B.; He, S.; Wu, B.; Zhu, P.; Luo, Q.; Gerwick, W.H.; et al. 5-Hydroxycyclopenicillone, a New β-Amyloid Fibrillization Inhibitor from a Sponge-Derived Fungus Trichoderma sp. HPQJ-34. *Mar. Drugs* **2017**, *15*, 260, doi:10.3390/MD15080260.
15. Liu, M.; Ohashi, M.; Hung, Y.-S.; Scherlach, K.; Watanabe, K.; Hertweck, C.; Tang, Y. AoiQ Catalyzes Geminal Dichlorination of 1,3-Diketone Natural Products. *J. Am. Chem. Soc.* **2021**, *143*, 7267–7271,

doi:10.1021/JACS.1C02868.

16. Nielsen, K.F.; Månsson, M.; Rank, C.; Frisvad, J.C.; Larsen, T.O. Dereplication of Microbial Natural Products by LC-DAD-TOFMS. *J. Nat. Prod.* **2011**, *74*, 2338–2348, doi:10.1021/NP200254T.
17. Bailey, A.M.; Cox, R.J.; Harley, K.; Lazarus, C.M.; Simpson, T.J.; Skellam, E. Characterisation of 3-methylorcinaldehyde synthase (MOS) in Acremonium strictum: first observation of a reductive release mechanism during polyketide biosynthesis{. *Chem. Commun.* **2007**, 4053–4055, doi:10.1039/b708614h.
18. Halo, L.M.; Marshall, J.W.; Yakasai, A.A.; Song, Z.; Butts, C.P.; Crump, M.P.; Heneghan, M.; Bailey, A.M.; Simpson, T.J.; Lazarus, C.M.; et al. Authentic Heterologous Expression of the Tenellin Iterative Polyketide Synthase Nonribosomal Peptide Synthetase Requires Coexpression with an Enoyl Reductase. *ChemBioChem* **2008**, *9*, 585–594, doi:10.1002/CBIC.200700390.
